# Image processing and AI techniques for climate change detection using remote sensing: a comprehensive review

**DOI:** 10.3389/frai.2026.1814362

**Published:** 2026-05-20

**Authors:** Anirudh Agarwal, Shreya Kumar, G. K. Rajini, Indragandhi Vairavasundaram

**Affiliations:** School of Electrical Engineering, Vellore Institute of Technology, Vellore, India

**Keywords:** change detection, climate change detection, deep learning (CNN, time-series), LULC change, performance metrics, remote sensing, satellite image processing

## Abstract

Climate change is accelerating spatially complex transformations in land, water, coasts, cryosphere, and ecosystems, creating a critical need for reliable, scalable, and timely monitoring based on Earth observation imagery. Conventional approaches that rely on sparse *in-situ* measurements, manual image interpretation, and simple spectral indices or thresholding often fail to capture subtle, heterogeneous, and multiscale changes, and they do not scale to today's multi-sensor, multi-temporal satellite archives. This review synthesizes image processing and AI techniques applied to optical, SAR, thermal, and hyperspectral remote sensing for climate change detection, covering classical change detection methods, machine learning classifiers, deep learning architectures (including Siamese and segmentation networks), spatio-temporal models for satellite image time series, and multi-sensor fusion, across application domains such as land use/land cover (LULC) and deforestation, hydrology and flooding, coastal and mangrove dynamics, cryospheric change, urban heat, ecosystems, and natural hazards. In addition, we analyze how these methods are evaluated using common performance metrics–Overall Accuracy (OA), precision, recall, F1-score, Intersection over Union (IoU), Kappa coefficient, and error measures such as RMSE–and discuss key challenges related to data quality and annotation, domain shift and generalization, computational and operational constraints, interpretability, and integration with climate and impact models. The distinctive contribution of this review is a unified method-application taxonomy that explicitly links algorithm families to specific climate monitoring tasks, a systematic comparison of reported performance metrics that clarifies trade-offs between techniques under different data and class-imbalance conditions, and a practical decision framework to guide researchers and practitioners in selecting appropriate image processing and AI approaches for given sensors, regions, and operational requirements, while outlining promising future directions such as foundation models, standardized benchmarks, and interoperable climate decision-support systems. Across the reviewed literature, deep learning approaches consistently demonstrate higher accuracy (e.g., improved IoU and F1-scores) in complex and heterogeneous environments, while classical methods remain effective for large-scale and data-scarce applications. However, significant gaps persist in model generalization across regions, availability of labeled datasets, and integration of multi-sensor time-series data.

## 1 Introduction

### 1.1 Climate change monitoring and the role of imaging

Climate change is altering the Earth system at unprecedented rates, affecting temperature, precipitation patterns, sea level, cryospheric extent, ecosystems, and human settlements in ways that are often spatially heterogeneous and non-linear. For science, policy, and adaptation planning, it is not sufficient to track global averages alone; decision-makers require spatially explicit, time-resolved information on where and how rapidly land cover, water bodies, coastlines, and urban areas are changing. Ground-based observation networks, while indispensable, remain too sparse and unevenly distributed to characterize these dynamics at regional to global scales, particularly in remote, data-poor, or politically sensitive regions.

In this context, satellite and airborne multi-sensor data have become a cornerstone of climate change monitoring because they provide synoptic, repeatable coverage over decades, with consistent measurements that support both long-term trend analysis and near-real-time event detection. The expanding constellation of optical, synthetic aperture radar (SAR), thermal, and hyperspectral sensors offers complementary perspectives on the land surface, oceans, and cryosphere, capturing signals related to vegetation condition, surface water extent, snow and ice cover, Land Surface Temperature (LST), and built-up expansion.

However, the volume, diversity, and temporal depth of modern remote sensing archives far exceed what can be analyzed through manual interpretation or simple threshold-based spectral indices. Subtle, spatially mixed, or gradually evolving climate-driven changes are easily overlooked by traditional techniques. Automated image processing and artificial intelligence (AI)-based methods are therefore essential to extract robust, scalable, and timely information from these data streams (Bône et al., 2023; Gu and Zeng, 2024; Shafique et al., 2022; Farooq and Manocha, 2024; Lu and Weng, 2020; Zhu et al., 2017). Such approaches enable continuous monitoring of climate-relevant changes, objective comparison across regions and time periods, and the integration of satellite-derived indicators into climate risk assessment and decision-support systems. Despite rapid advancements in remote sensing and AI-based change detection, existing studies often remain fragmented, focusing either on specific techniques or isolated application domains. There is a lack of integrated frameworks that systematically compare methods across different climate processes while accounting for variations in sensor data, spatial scale, and temporal dynamics. This gap limits the ability of researchers and practitioners to select appropriate techniques for real-world climate monitoring tasks.

The evolution from classical image processing methods to machine learning and deep learning approaches has been driven by several key factors. The increasing availability of large-scale satellite datasets, improvements in computational power, and the need to capture complex, non-linear environmental patterns have significantly influenced methodological development. While early approaches relied on simple spectral thresholds and index-based differencing, modern methods leverage data-driven learning to extract hierarchical spatial and temporal features. This transition has enhanced the ability to detect subtle and heterogeneous changes, enabling more accurate and scalable climate monitoring across diverse regions and sensor types. However, this progression also introduces new challenges related to data requirements, model interpretability, and generalization across regions, highlighting the need for balanced and application-specific method selection.

The techniques reviewed in this study are applicable to a wide range of climate change manifestations, including land use and land cover transitions, deforestation and land degradation, urban expansion and urban heat island effects, hydrological changes such as floods and droughts, coastal erosion and shoreline shifts, cryospheric dynamics including glacier retreat and snow cover variability, as well as ecosystem and biodiversity changes. By explicitly linking image analysis methods to these climate processes, the review highlights how advances in remote sensing and AI contribute to improved monitoring, attribution, and assessment of climate impacts across diverse environmental systems. This categorization also provides a structured basis for organizing the application domains discussed in subsequent sections.

### 1.2 Conventional climate monitoring challenges

Conventional approaches to climate monitoring face several structural limitations that restrict their ability to capture the full complexity of ongoing environmental change. *In-situ* observation networks, including weather stations, river gauges, and flux towers, provide high-quality point measurements but are sparsely and unevenly distributed. Large gaps persist across much of the Global South, high-latitude regions, and remote ecosystems. Moreover, these networks often lack the spatial resolution required to resolve localized land use, hydrological, or coastal changes, and many stations do not operate continuously over multi-decadal periods.

Non-imaging climate indicators derived from reanalysis products or coarse-resolution gridded datasets are valuable for large-scale assessments but tend to smooth fine-scale spatial heterogeneity. This limitation complicates the attribution of observed climate trends to specific landscape transformations, such as localized deforestation fronts, urban expansion corridors, or small reservoirs and wetlands. When satellite data is employed using traditional workflows, analysis frequently relies on manual or semi-manual interpretation and simple spectral indices, for example, normalized difference vegetation index (NDVI) or normalized difference water index (NDWI) differencing with fixed thresholds. Such approaches are sensitive to illumination conditions, seasonal variability, and atmospheric effects, and they often fail to detect subtle, gradual, or spectrally complex changes, including land degradation, shifts in land management practices, mixed pixels in peri-urban environments, or overlapping signals arising from concurrent drought and land use change (Chughtai et al., 2021; Lu and Weng, 2020; Hussain et al., 2013; Lu et al., 2004; Singh, 1989; Coppin et al., 2004).

In addition, these conventional workflows are difficult to scale to contemporary Earth observation data volumes. Modern satellite programs generate petabytes of multi-sensor, multi-temporal imagery, rendering manual interpretation and rule-based processing increasingly time-consuming and inconsistent across regions and analysts. As a result, traditional climate monitoring systems frequently deliver delayed, coarse, or incomplete information, limiting their effectiveness for near-real-time climate risk assessment, early warning, and the iterative evaluation of adaptation and mitigation measures.

### 1.3 Overview of remote sensing and image data

Remote sensing for climate monitoring draws on a family of complementary imaging systems that differ in spatial resolution, revisit frequency, spectral coverage, and sensitivity to surface and atmospheric conditions (Lu and Weng, 2020; Gomez-Chova et al., 2020; Richards and Jia, 2013; Camps-Valls et al., 2014; Zhu et al., 2017). Optical multispectral sensors such as Landsat, Sentinel-2, and MODIS measure reflected solar radiation in visible to shortwave infrared bands and are widely used to derive vegetation indices, water indices, snow and ice maps, and built-up indicators. These sensors provide rich spectral information and long historical archives, but their utility is constrained by cloud cover and atmospheric effects, and individual missions often involve trade-offs between spatial detail and temporal frequency (Fu et al., 2024; Richards and Jia, 2013).

SAR missions, exemplified by Sentinel-1, actively transmit microwave signals and record the backscattered energy from the Earth's surface. SAR systems enable day-and-night, all-weather observations and are particularly valuable for monitoring floods, soil moisture, surface roughness, and structural changes in vegetation and urban areas. However, SAR data interpretation is more complex than that of optical imagery due to the presence of speckle noise and the strong dependence of backscatter on imaging geometry, surface roughness, and polarization.

Thermal infrared sensors capture emitted radiation related to LST and surface energy balance, enabling the analysis of urban heat islands, drought stress, and heatwave dynamics. These data are essential for linking land surface processes with climate extremes, although thermal observations are often available at coarser spatial resolution than visible and near-infrared imagery. Hyperspectral instruments acquire contiguous narrow spectral bands across wide wavelength ranges, supporting fine discrimination of vegetation species, soil and mineral properties, and water quality characteristics. While hyperspectral data offer unique analytical capabilities, they are associated with larger data volumes, more demanding preprocessing requirements, and typically smaller spatial coverage compared to multispectral sensors (Camps-Valls et al., 2014; Ghamisi et al., 2017; Gong et al., 2019). This diversity of sensors allows climate monitoring systems to be tailored to specific processes and spatial scales (Gomez-Chova et al., 2020; Camps-Valls et al., 2014; Zhu et al., 2017). For example, Sentinel-2 and Landsat data are commonly combined for detailed LULC change mapping, MODIS products support global and long-term trend analysis, Sentinel-1 enables cloud-robust monitoring of floods and wetlands, thermal infrared observations provide information on LST dynamics, and hyperspectral data support targeted studies of ecosystems and biogeochemical processes. A summary of the main characteristics of commonly used remote sensing image data for climate monitoring is provided in Table 1.

**Table 1:** Representative satellite remote sensing sensors used for climate-relevant monitoring.

Sensor	Type	Description
Landsat-8/9	Optical MS + TIR	30 m VNIR/SWIR and 100 m thermal infrared observations with a 16-day revisit, supporting long-term LULC change analysis, deforestation monitoring, lake and glacier area mapping, and LST estimation.
Sentinel-2	Optical MS	10–20 m multispectral imagery with a 5-day revisit and red-edge bands, enabling high-resolution land cover mapping, vegetation condition assessment, and coastal and inland water monitoring.
MODIS	Optical MS + TIR	Moderate-resolution (250–1,000 m) daily global observations across VNIR, SWIR, and thermal bands, widely used for global vegetation dynamics, snow cover, fire detection, and LST analysis.
Sentinel-1	C-band SAR	All-weather, day-and-night radar imaging at approximately 10 m resolution with VV/VH or HH/HV polarizations, enabling flood mapping, soil moisture estimation, ice dynamics monitoring, and surface deformation analysis (Bhatt et al., 2022; Ban and Yousif, 2016).
EnMAP / PRISMA	Hyperspectral	Hyperspectral imaging with over 100 contiguous VNIR-SWIR bands at 30 m resolution, supporting detailed retrieval of vegetation traits, ecosystem condition, soil properties, and water quality parameters.

### 1.4 Benefits of AI and image processing for climate applications

Advanced AI-based methods fundamentally extend what can be extracted from remote sensing data for climate applications, both in terms of accuracy and scale (Gu and Zeng, 2024; Shafique et al., 2022; Farooq and Manocha, 2024; Bai et al., 2023; Zhu et al., 2017). By learning complex, non-linear relationships between spectral, spatial, and temporal patterns and underlying surface processes, machine learning and deep learning models consistently outperform simple index-based thresholds and rigid rule sets (Gu and Zeng, 2024; Shafique et al., 2022; Bai et al., 2023; Chen et al., 2020; Peng et al., 2019). This advantage is particularly evident in heterogeneous landscapes, mixed pixels, and regions where climate variability and human activities interact in non-trivial ways.

A key benefit of AI-driven approaches is the automation of large-scale monitoring pipelines (Pickens et al., 2025; Shafique et al., 2022; Bai et al., 2023; Yu et al., 2024). Once trained, these models enable continuous or near-real-time analysis over entire regions or the globe, a task that would be infeasible using manual interpretation or *ad hoc*, rule-based workflows. Automated methods are especially effective at detecting fine-scale or spatially fragmented changes, such as small deforestation clearings, narrow corridors of urban expansion, localized flood inundation, or subtle forms of vegetation degradation that are often missed by conventional techniques.

Modern architectures also support multi-sensor fusion, allowing optical, SAR, thermal, and ancillary datasets to be integrated within a unified analytical framework (Miller et al., 2024; Fu et al., 2024; Yu et al., 2024; Ghamisi et al., 2017). This fusion improves robustness under challenging conditions, including persistent cloud cover, variable illumination, or partial data loss. In parallel, time-series and spatio-temporal models explicitly exploit temporal dynamics, enabling the separation of genuine long-term change from seasonal cycles, interannual variability, or short-lived disturbances.

**Figure 1:**
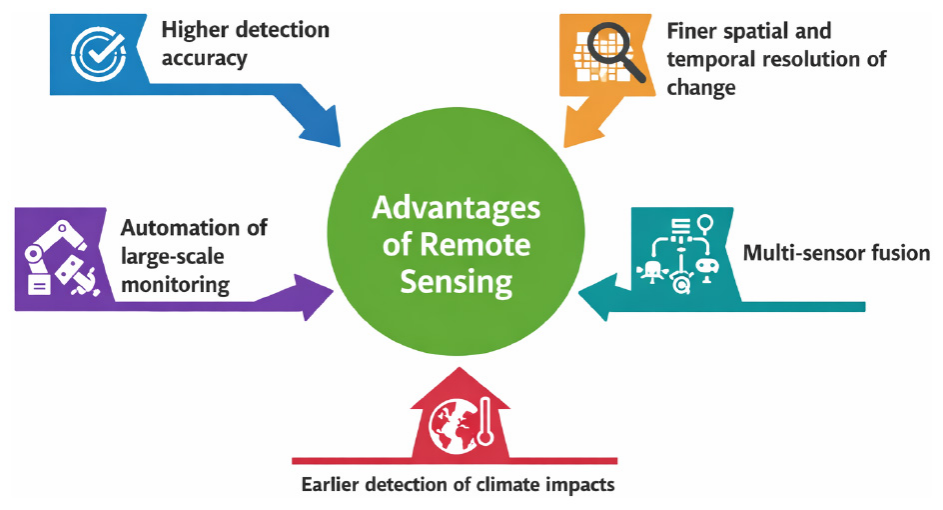
Advantages of remote sensing for climate monitoring (Bhatt et al., 2022; Ban and Yousif, 2016).

At the system level, these methodological advances translate into earlier and more reliable detection of climate impacts, such as emerging deforestation fronts, intensifying urban heat islands, or shifting flood regimes. As a result, AI-enabled image analysis enhances climate risk assessment, supports targeted adaptation and mitigation strategies, and provides objective, spatially explicit evidence to inform policy design, implementation, and evaluation. The advantages of remote sensing for climate monitoring are illustrated in Figure 1.

### 1.5 Scope and structure of the review

This review focuses specifically on climate change detection and monitoring using data-driven approaches applied to satellite and other satellite data, rather than on general climate modeling or sensor hardware design (Gu and Zeng, 2024; Shafique et al., 2022; Farooq and Manocha, 2024; Chughtai et al., 2021; Bai et al., 2023; Rewhel et al., 2023). The scope is intentionally centered on methods that operate directly on image data to detect, characterize, and quantify spatial and temporal changes associated with climate processes and impacts.

The review is organized around a dual taxonomy that links technique types–ranging from classical image processing and conventional machine learning to deep learning and satellite image time-series models–to specific climate application domains (Shafique et al., 2022; Farooq and Manocha, 2024; Bai et al., 2023; Yu et al., 2024; Lu and Weng, 2020). These domains include LULC change and deforestation, hydrology and flooding, coastal and mangrove dynamics, cryospheric change, urban heat, ecosystems and biodiversity, and natural hazards. Beyond cataloging methods and case studies, particular emphasis is placed on performance evaluation, synthesizing how different approaches are assessed using metrics such as OA, precision and recall, F1-score, IoU, Kappa coefficient, and error statistics. Unlike existing surveys that primarily focus on either methodological developments or specific application domains, this review provides an integrated method-application perspective by explicitly linking AI techniques to distinct climate related processes. Furthermore, it introduces a comparative synthesis of performance metrics across studies and proposes a practical decision framework that guides method selection based on sensor type, data availability, and operational constraints. This dual analytical and decision-oriented approach distinguishes the present work from prior reviews.

**Figure 2:**
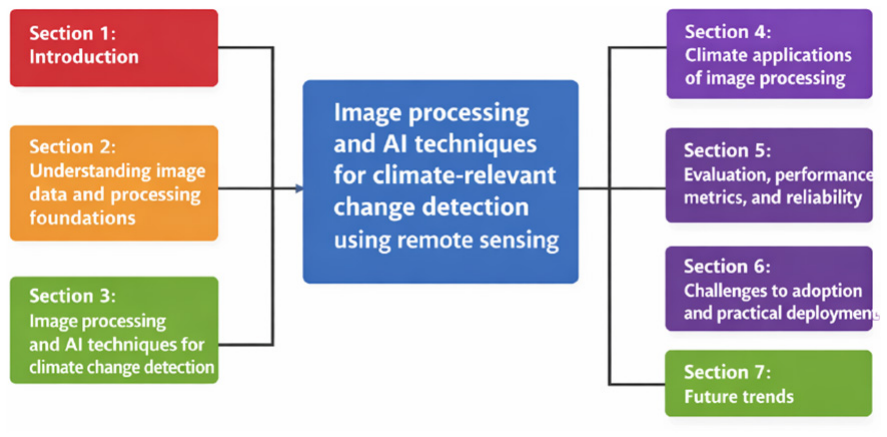
Structure of the review paper.

Distinct from earlier, broader surveys of remote sensing or environmental AI, this work provides an integrated classification matrix that explicitly maps algorithm families to climate monitoring tasks and data regimes (Gu and Zeng, 2024; Shafique et al., 2022; Bai et al., 2023; Rewhel et al., 2023; Zhu et al., 2017). In addition, it develops a practical decision framework to help researchers and practitioners select appropriate methods under varying constraints related to sensor type, spatial and temporal scale, computational resources, and operational requirements. The overall organization of this review and the relationship between major sections are illustrated in Figure 2.

### 1.6 Review methodology

The literature reviewed in this paper was identified through a structured search of major scientific databases, including IEEE Xplore, ScienceDirect, SpringerLink, MDPI, and Wiley, complemented by targeted queries of open repositories such as arXiv and general scholarly search engines (Gu and Zeng, 2024; Shafique et al., 2022; Farooq and Manocha, 2024; Fu et al., 2024; Bai et al., 2023). The search focused primarily on studies published between 2015 and 2025, a period that captures the rapid growth of deep learning techniques and the expansion of large-scale, multi-sensor remote sensing archives, while also incorporating a limited number of earlier foundational works on classical change detection where necessary for context (Shafique et al., 2022; Miller et al., 2024; Fu et al., 2024; Yu et al., 2024; Zhu et al., 2017).

Combinations of keywords such as *climate change, remote sensing, satellite imagery, image processing, change detection, LULC, flood mapping, glacier retreat, urban heat island, machine learning*, and *deep learning* were applied to retrieve potentially relevant articles. Inclusion criteria required that a study (i) employ Earth observation data (optical, SAR, thermal, or hyperspectral), (ii) address a clearly climate application such as LULC dynamics, water and flood monitoring, coastal or cryospheric change, temperature-related impacts, ecosystems, or climate-driven hazards, and (iii) either report or substantively discuss performance evaluation, whether qualitative or quantitative (Farooq and Manocha, 2024; Chughtai et al., 2021; Bai et al., 2023; Ghaderpour and Vujadinovic, 2020).

**Figure 3:**
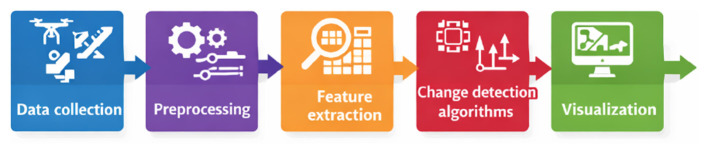
Methodology pipeline for climate change detection.

Excluded from the review were studies focused solely on climate or weather modeling without explicit image-level analysis, purely social or perceptual studies of climate imagery, and sensor or platform engineering papers that did not develop or evaluate image processing or AI methods for environmental change detection (Gu and Zeng, 2024; Shafique et al., 2022). After removing duplicates and screening titles and abstracts, full-text review was conducted to ensure alignment with these criteria. The final set of papers was then organized according to the method-application taxonomy that underpins the structure of this review (Shafique et al., 2022; Bai et al., 2023; Yu et al., 2024). The overall methodological workflow adopted in this review, from data acquisition through preprocessing, feature extraction, change detection, and visualization, is summarized in Figure 3.

## 2 Understanding image data and processing foundations

Remote sensing-based climate monitoring relies on a diverse set of imaging systems and a sequence of processing steps that transform raw measurements into physically meaningful variables and change indicators. This section outlines the main types of satellite imagery, the key preprocessing operations required for robust analysis, and the climate related signals that can be extracted from these data (Fu et al., 2024; Lu and Weng, 2020; Richards and Jia, 2013; Zhu et al., 2017).

### 2.1 Remote sensing imagery types and characteristics

Remote sensing imagery used in climate monitoring can be broadly categorized into optical multispectral, SAR, thermal infrared, and hyperspectral data, each providing complementary information about surface processes. Optical sensors such as Landsat and Sentinel-2 capture reflected radiation and are widely used for vegetation, land cover, and water mapping. However, their performance is constrained by cloud cover and atmospheric effects.

SAR systems, such as Sentinel-1, overcome these limitations by providing all-weather, day-and-night observations, making them particularly suitable for flood detection, soil moisture estimation, and structural monitoring of vegetation and urban areas. Thermal sensors provide critical information on land surface temperature and energy balance, supporting studies of heatwaves and urban heat islands, although typically at coarser spatial resolution. Hyperspectral data offer detailed spectral information for fine discrimination of vegetation and soil properties but involve higher data complexity and processing requirements.

Importantly, these sensor characteristics directly influence method selection. Optical data are well suited for spectral index-based and deep learning classification approaches, while SAR data require models robust to speckle noise and are often used in flood and structural change detection. High temporal resolution datasets favor time-series and spatio-temporal models, whereas high spatial resolution imagery supports deep learning-based segmentation for fine-scale mapping.

The choice of image processing or AI technique is strongly influenced by sensor characteristics. For instance, SAR data, which are affected by speckle noise and complex backscatter properties, often require robust preprocessing and noise-resistant models, while optical data are more suitable for spectral index-based and convolutional approaches. Hyperspectral data, with their high dimensionality, benefit from advanced feature extraction techniques such as deep neural networks. Similarly, datasets with high temporal resolution enable the use of time-series and spatio-temporal models, whereas high spatial resolution imagery is more compatible with segmentation-based deep learning architectures. This interdependence highlights the need to jointly consider sensor properties and methodological design in climate change detection.

### 2.2 Preprocessing and data quality issues

Before satellite observations can be reliably used for climate change detection and quantitative analysis, it must undergo a series of preprocessing steps that correct for sensor, geometric, and atmospheric effects and ensure consistency across space, time, and sensors (Richards and Jia, 2013; Coppin et al., 2004; Foody, 2002).

Radiometric and atmospheric corrections convert raw digital numbers to calibrated surface reflectance or radar backscatter, preventing false change detection caused by variations in illumination and atmospheric conditions (Richards and Jia, 2013; Coppin et al., 2004). Geometric correction and co-registration align multi-temporal images to a common spatial framework, which is essential for pixel-level and time-series analysis, as even small misalignments can introduce significant errors (Coppin et al., 2004; Hussain et al., 2013).

Cloud and shadow masking, gap filling, and compositing are particularly important for optical imagery, where missing or contaminated observations can bias results (Fu et al., 2024; Chen and Zhang, 2014). In multi-sensor workflows, normalization and harmonization ensure consistency across datasets with different spectral responses and spatial resolutions, enabling reliable long-term analysis (McGregor et al., 2024; Babitha et al., 2024; Ghamisi et al., 2017).

These preprocessing steps are not merely technical requirements but directly influence model performance, as residual noise, misalignment, or calibration inconsistencies can propagate into false detections, especially in sensitive climate applications. The choice and quality of preprocessing directly affect the reliability of subsequent image processing and AI-based change detection methods.

### 2.3 Climate signals in images

Processed remote sensing data are transformed into climate related indicators that capture environmental dynamics and serve as proxies for climate processes (Fu et al., 2024; Chen and Zhang, 2014; Roy et al., 2020). These indicators are typically expressed as spectral indices, classifications, or continuous variables derived from optical, SAR, and thermal imagery.

Vegetation indices such as NDVI and EVI are widely employed for monitor ecosystem health, drought stress, land degradation, and phenological changes over time (Chen and Zhang, 2014; Roy et al., 2020). Water-related indices, including NDWI and SAR-based classifications, enable mapping of surface water dynamics, floods, and wetlands, supporting hydrological monitoring and disaster assessment (Khalil, 2025; Fang et al., 2021).

Thermal observations provide land surface temperature (LST) estimates, which are critical for analyzing urban heat islands, heatwaves, and surface energy balance, while snow and ice products derived from optical and SAR data support monitoring of glacier dynamics and cryospheric change (Vasquez et al., 2024; Shekhar et al., 2022). Built-up indices and land cover classifications capture urban expansion and its interaction with climate, hydrology, and environmental quality (Vahid and Aly, 2025; King et al., 2021; Roy et al., 2020).

**Figure 4:**
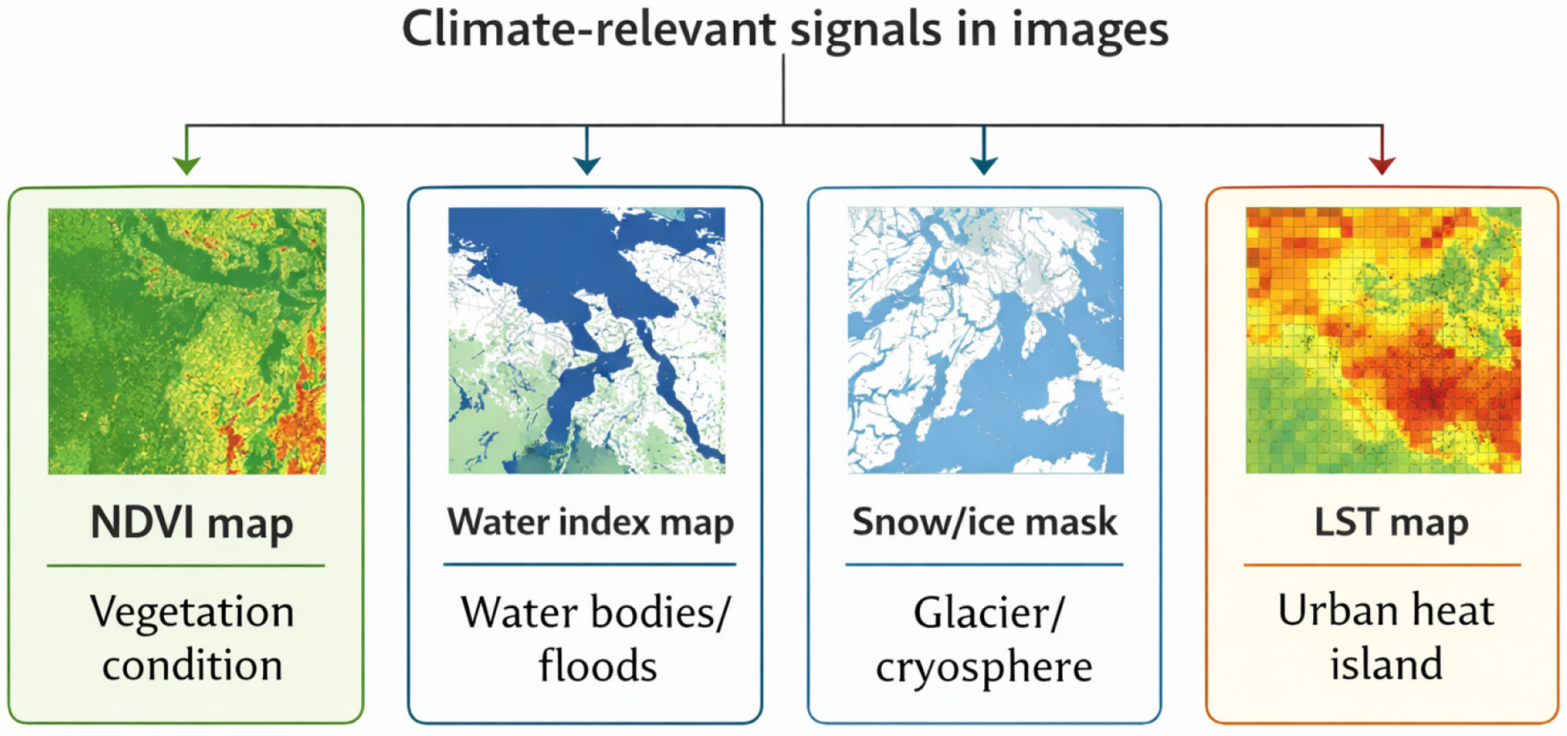
Climate signals derived from remote sensing imagery.

These derived signals form the critical link between raw imagery and physically meaningful climate variables, enabling integration with environmental models, risk assessment frameworks, and decision-support systems (Vahid and Aly, 2025; Lu and Weng, 2020). The choice of these indicators directly influences the selection of learning-based models, as different variables require different modeling approaches. Representative climate-relevant image-derived signals and their typical interpretations are illustrated in Figure 4.

## 3 Image processing and AI techniques for climate change detection

This section reviews the main classes of image processing and AI techniques used to detect, characterize, and monitor climate related environmental change from satellite observations. Building on the data foundations described in Section 2, it examines classical change detection approaches, machine learning methods, deep learning architectures, and spatio-temporal models, highlighting their respective strengths, limitations, and methodological characteristics (Shafique et al., 2022; Farooq and Manocha, 2024; Bai et al., 2023; Lu and Weng, 2020).

### 3.1 Design criteria for climate related image analysis

Designing image analysis methods for climate change detection imposes multiple, partly competing requirements that go beyond standard image classification or segmentation tasks. First, algorithms must accommodate a wide range of spatial and temporal scales. Methods should perform effectively on high-resolution imagery for local processes such as urban expansion or small-patch deforestation, while also remaining applicable to coarser-resolution sensors used for regional to global trend monitoring over years to decades. Importantly, they must preserve sensitivity to both abrupt events and gradual, cumulative shifts.

Second, climate-oriented image analysis demands strong robustness to noise and observation variability. This includes resilience to clouds and shadows in optical imagery, residual atmospheric effects, sensor calibration differences, seasonal and interannual phenological variability, and speckle noise in SAR data. Robust methods are essential to ensure that detected changes correspond to genuine environmental signals rather than artifacts of acquisition conditions or preprocessing.

Third, practical climate applications increasingly require multi-sensor compatibility and data fusion capabilities. Effective approaches should be able to ingest or integrate optical, SAR, thermal, and potentially hyperspectral imagery, as well as ancillary geospatial data. This requires handling differences in spatial resolution, viewing geometry, and revisit frequency while exploiting the complementary information content of diverse sensors.

Fourth, because climate assessments and policy decisions rely on scientific credibility and transparency, image analysis models should offer a degree of interpretability and uncertainty characterization. Users must be able to understand which features, regions, or temporal patterns drive a detection, assess the confidence associated with outputs, and relate algorithmic results to physically meaningful variables and processes.

Finally, operational deployment places constraints on computational cost and scalability. Methods must be capable of processing large historical archives and near-real-time data streams using reasonable hardware resources. Scalable architectures, efficient training and inference workflows, and reproducible implementations are critical for sustained monitoring programs and long-term climate services (Gu and Zeng, 2024; Shafique et al., 2022; Yu et al., 2024; Zhu et al., 2017).

### 3.2 Classical image processing and change detection methods

Early, and still widely used, approaches to change detection in remote sensing rely on classical image processing techniques that operate directly on pixel values or simple transformations thereof (Hussain et al., 2013; Lu et al., 2004; Singh, 1989; Coppin et al., 2004). One of the most common techniques is index differencing, in which vegetation or water indices such as the NDVI or the NDWI are computed for two acquisition dates and subtracted. Pixels exceeding a predefined positive or negative threshold are flagged as change. This approach performs well for pronounced changes, such as clear-cut various land cover classification and change detection tasks (De and Barman, 2021; Bhatt et al., 2022; Fang et al., 2021), but it is sensitive to seasonal variability, illumination differences, and residual atmospheric effects.

Band ratioing and image regression extend this idea by normalizing spectral relationships between dates or explicitly modeling the expected pixel-wise relationship under no-change conditions. Changes are then detected by thresholding regression residuals. These methods can improve robustness to illumination and sensor differences in some contexts, but their performance still depends strongly on appropriate model specification and threshold selection.

Principal Component Analysis (PCA)-based change detection stacks multi-temporal images and uses components associated with temporal variance to highlight change-related information. PCA can capture complex, multi-band spectral shifts that are difficult to detect using single-index approaches, but the resulting components are often difficult to interpret in physical terms, which limits their usefulness for process-based climate analysis.

Change Vector Analysis (CVA) represents spectral reflectance as vectors in multi-dimensional feature space and quantifies change as the magnitude, and sometimes direction, of the vector difference between dates. This enables multi-band change detection and can distinguish between different types of change, such as vegetation loss vs. vegetation gain. However, CVA remains sensitive to noise and requires careful selection of thresholds and feature spaces (Bovolo and Bruzzone, 2007; Coppin et al., 2004).

Post-classification comparison follows a different strategy: each image is independently classified into land-cover classes, and change is inferred by comparing class labels between dates. This approach is intuitive and allows class-specific change analysis, but it is heavily dependent on classification accuracy at each time step and is prone to error propagation when classification errors accumulate across dates (Lu et al., 2004; Coppin et al., 2004; Foody, 2002).

Object-based image analysis (OBIA), also referred to as geographic object-based image analysis (GEOBIA), segments images into meaningful objects, such as patches or parcels, prior to change detection. By incorporating shape, texture, and contextual information, object-based methods often outperform pixel-based techniques in high-resolution imagery. However, they require careful segmentation and parameter tuning, and their transferability across regions and sensors is limited.

Overall, classical change detection techniques are relatively simple and computationally efficient, making them attractive for long time series, operational monitoring, and data-poor settings. Nevertheless, they generally struggle with subtle, noisy, or highly heterogeneous climate-driven changes and require extensive manual calibration to generalize reliably across regions and time periods (Hussain et al., 2013; Lu et al., 2004; Coppin et al., 2004). However, despite their simplicity and computational efficiency, classical change detection methods exhibit limited robustness in heterogeneous and dynamically changing landscapes. Their performance is highly sensitive to threshold selection, seasonal variability, and atmospheric effects, which can lead to significant omission and commission errors. Furthermore, their reliance on linear transformations and handcrafted indices restricts their ability to capture complex, non-linear relationships inherent in climate-driven processes such as gradual land degradation, mixed land-use transitions, and subtle ecosystem changes. As a result, their applicability is often limited to large, high-contrast changes or well-controlled datasets. A concise comparison of representative classical change detection methods, highlighting their strengths, limitations, and typical applications, is provided in Table 2.

**Table 2:** Comparison of representative classical image processing methods for change detection.

Technique	Main idea	Pros	Cons
NDVI / index differencing	Subtract spectral indices between dates	Simple, fast, intuitive; effective for strong signals	Sensitive to seasonality and atmosphere; single-index dependence
Band ratioing / regression	Normalize or model inter-band relationships	Reduces illumination and sensor differences	Limited for complex spectra; threshold choice critical
PCA-based change detection	Use principal components capturing temporal variation	Captures multi-band change; data-driven	Components difficult to interpret; may mix noise and signal
Change Vector Analysis (CVA)	Magnitude and direction of spectral change vectors	Multi-band; can separate change types	Sensitive to noise; requires careful thresholding
Post-classification comparison	Compare independently classified maps	Class-specific change; intuitive interpretation	Error propagation; depends on classification accuracy
Object-based approaches (OBIA)	Segment images, then classify or compare objects	Uses shape and context; effective for high-resolution data	Segmentation sensitive; higher workflow complexity

### 3.3 Machine learning methods

Machine learning methods extend classical image processing approaches by learning data-driven decision rules from labeled examples, typically using engineered feature vectors derived from one or more images. In supervised settings, algorithms such as Support Vector Machines (SVM), Random Forests (RF), gradient boosting methods (e.g., XGBoost, LightGBM), and other ensemble learners are widely used for LULC classification. These classifications can then be compared across dates for post-classification change detection, or models can be trained directly on multi-temporal feature stacks to identify change vs. no-change or specific transition types (Gu and Zeng, 2024; Chughtai et al., 2021; Vahid and Aly, 2025; Tesfaye et al., 2024).

RF and gradient boosting models have their popularity stem from their robustness to noisy and correlated features, ability to model non-linear relationships, and the availability of internal measures of feature importance that provide a degree of interpretability. These properties make them attractive for climate monitoring tasks where training data may be imperfect and explanatory insight is often required alongside predictive accuracy (Yang et al., 2024; Gu and Zeng, 2024; Vahid and Aly, 2025).

Unsupervised and semi-supervised methods are also employed when labeled data are scarce or unavailable. Techniques such as K-means clustering, Gaussian mixture models, and self-organizing maps have been used to identify anomalous change regions, group pixels or objects with similar temporal behavior, or provide an initial segmentation that can be refined through manual interpretation or limited labeling (Ghaderpour and Vujadinovic, 2020; Lu and Weng, 2020).

A central component of machine learning-based change detection is feature engineering. Rather than relying solely on raw spectral bands, practitioners typically construct rich feature sets that include spectral indices (e.g., NDVI, EVI, NDWI, bare soil and built-up indices), temporal statistics (means, medians, percentiles, and seasonal composites), textural metrics such as gray-level co-occurrence matrix (GLCM) features that capture local heterogeneity, and topographic variables including elevation, slope, and aspect. In some studies, climate variables, soil properties, or proximity measures are also incorporated to provide additional contextual information (Fu et al., 2024; Vahid and Aly, 2025; Chen and Zhang, 2014).

For SAR-based analyses, commonly used features include backscatter intensity in different polarizations, temporal analysis coherence, and speckle-filtered statistics. By combining such handcrafted features, machine learning methods can capture key aspects of land cover, vegetation condition, and hydrological state relevant to climate monitoring, while remaining relatively transparent and less data-intensive than deep learning approaches. However, their performance and transferability depend strongly on feature selection and data quality, and they may struggle in very high-dimensional, multi-sensor, or strongly class-imbalanced settings without careful tuning, feature selection, or sampling strategies. While machine learning approaches improve classification accuracy and provide greater flexibility compared to classical methods, they remain strongly dependent on the quality and representativeness of input features and training data. Feature engineering plays a critical role, and suboptimal feature selection can significantly degrade performance. In addition, these models often struggle with generalization across regions, sensors, and temporal conditions due to domain shift. Although ensemble methods such as Random Forest offer some robustness, their limited capacity to explicitly model spatial context and temporal dynamics restricts their effectiveness in complex, multi-temporal climate monitoring scenarios.

### 3.4 Deep learning methods

Deep learning methods learn hierarchical feature representations directly from image data and have become state of the art for many climate remote sensing tasks. Convolutional Neural Networks (CNNs) are widely used for various types of environmental change, either in patch-based form, where image chips are classified independently, or as fully convolutional architectures that generate dense prediction maps over entire scenes (Shafique et al., 2022; Bai et al., 2023; Zhu et al., 2017).

Segmentation-oriented networks, such as U-Net and its variants, are particularly effective for producing pixel-wise maps of deforestation, urban areas, flood water, snow cover, and shoreline position. Their encoder–decoder structure, combined with skip connections, allows them to capture both fine-scale texture and broader spatial context, which is essential for delineating complex and fragmented climate-driven patterns (VanExel et al., 2025; Chen et al., 2020; Daudt et al., 2018; Chen et al., 2016). For explicit change detection, Siamese network architectures take two images from different times as input, process them through shared feature extractors, and compare the resulting representations to infer change or no-change. This design naturally exploits relative information between dates and has been implemented using CNN backbones, U-Net-like encoders, and increasingly transformer-based architectures (Chen et al., 2020; Daudt et al., 2018).

Beyond single-sensor inputs, deep learning models are increasingly designed for multi-modal and multi-sensor fusion. Parallel network branches may ingest optical, SAR, thermal, or ancillary data streams, with intermediate or late fusion layers combining complementary information. Such designs improve robustness under cloud cover and variable acquisition conditions and enhance sensitivity to both structural and radiometric changes. When sufficient labeled data and computational resources are available, deep architectures typically achieve higher accuracy and better generalization than classical image processing or shallow machine learning approaches. However, they are also more data-hungry and less transparent, requiring careful regularization, data augmentation, and validation strategies to mitigate overfitting and support reliable deployment in climate monitoring contexts (Shafique et al., 2022; Bai et al., 2023; Zhu et al., 2017). Despite their superior performance and ability to learn hierarchical spatial and spectral representations, deep learning methods face several practical challenges that limit their widespread adoption. These models are highly data-intensive and require large volumes of accurately labeled training data, which are often scarce in many climate applications. Additionally, their computational demands for training and inference can be substantial, posing constraints for operational deployment. Another key limitation is their limited interpretability, as deep models often function as black boxes, making it difficult to explain predictions in physically meaningful terms. Furthermore, their performance can degrade significantly under domain shift, requiring careful validation and adaptation when applied to new regions or sensor types.

### 3.5 Time-series and spatio-temporal models

Many climate processes manifest not merely as differences between two acquisition dates, but as trajectories unfolding over months to decades, motivating models that explicitly capture spatio-temporal dynamics. Convolutional Long Short-Term Memory (ConvLSTM) networks extend standard LSTMs by replacing internal matrix multiplications with convolutions, enabling the modeling of image sequences while preserving spatial structure (Miller et al., 2024; He et al., 2024). These models have been applied to tasks such as capturing gradual and long-term environmental changes (Miller et al., 2024; Fu et al., 2024; He et al., 2024).

Temporal CNNs apply one-dimensional convolutions along the temporal axis, either at the pixel level or in combination with spatial convolutions, to extract temporal patterns such as trends, seasonal cycles, and abrupt breaks. Compared with recurrent architectures, temporal CNNs are often easier to train and can be scaled efficiently to large image time series (Miller et al., 2024; Fu et al., 2024). Transformer-based models adapted for remote sensing treat time steps and/or spatial patches as tokens and use self-attention mechanisms to learn long-range temporal and spatial dependencies simultaneously, making them well suited to irregular observation schedules and heterogeneous multi-sensor sequences (Yu et al., 2024; He et al., 2024).

**Figure 5:**
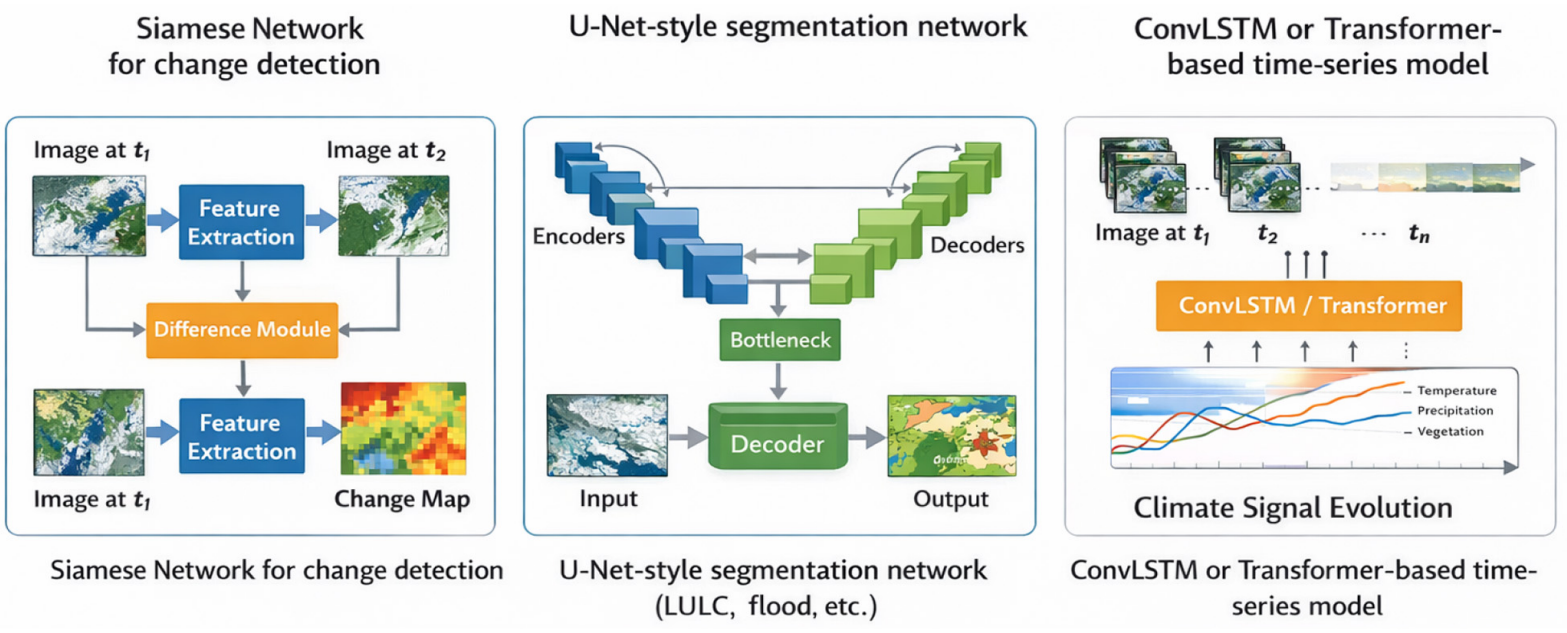
Representative deep learning model architectures for climate change detection.

More classical recurrent networks, including LSTM and Gated Recurrent Unit (GRU) architectures, remain in use, particularly when inputs consist of pre-extracted features such as vegetation index time series rather than raw imagery. These lighter-weight models can be effective for capturing temporal behavior when computational resources or labeled data are limited (Miller et al., 2024). Across architectures, explicit modeling of temporal context improves the ability to distinguish persistent, climate-driven change from short-term variability or noise and supports earlier detection of emerging impacts. At the same time, spatio-temporal models place increased demands on data preprocessing, including consistent co-registration, gap handling, and harmonization across sensors and long observation periods (Miller et al., 2024; Fu et al., 2024; Yu et al., 2024). Although spatio-temporal models significantly enhance the ability to capture dynamic environmental processes and distinguish long-term trends from short-term variability, they introduce additional complexity in data preparation and model training. These approaches require consistent time-series datasets, robust gap-filling strategies, and careful handling of missing or irregular observations. Moreover, their increased computational complexity and sensitivity to temporal inconsistencies can limit scalability and operational applicability in large-scale climate monitoring systems. Representative deep learning architectures used for change detection and spatio-temporal modeling are illustrated in Figure 5.

### 3.6 Comparative synthesis of techniques

Over the past two decades, methods for climate change detection have evolved from simple index-based and post-classification approaches toward increasingly sophisticated machine learning and deep learning architectures, each occupying a distinct niche in terms of data requirements, scalability, and interpretability (Shafique et al., 2022; Bai et al., 2023; Zhu et al., 2017). Classical image processing methods remain attractive for long historical time series, data-sparse regions, and applications where changes are large and spectrally clear, such as complete forest clearing or open-water expansion, because they are simple, transparent, and computationally inexpensive. However, they are less effective for subtle, noisy, or highly heterogeneous changes and often require scene-specific tuning.

Traditional machine learning methods based on engineered features and algorithms such as Random Forests or SVMs offer a balance between performance and interpretability. They perform well in medium-sized datasets and multi-source settings where spectral, textural, and topographic features can be explicitly designed, and they typically outperform purely classical techniques on complex LULC and hazard-mapping tasks. At the same time, they may struggle to fully exploit very high-dimensional image stacks or long, dense time series without careful feature selection and sampling strategies (Gu and Zeng, 2024; Vahid and Aly, 2025; Tesfaye et al., 2024).

Deep learning methods, including CNN-based segmentation, Siamese change detection networks, and spatio-temporal architectures such as ConvLSTM and transformers, provide the highest accuracy and flexibility in many recent studies, particularly for high-resolution, multi-sensor, and time-series data. These gains come at the cost of increased demands for labeled data, computational resources, and careful attention to generalization, and they often require additional effort to improve interpretability and robustness across regions and sensors (Shafique et al., 2022; Yu et al., 2024; Zhu et al., 2017). In practice, method choice is dictated by sensor availability, spatial and temporal scale, label budgets, and operational constraints, and hybrid workflows that combine classical preprocessing, machine-learned feature extraction, and deep models are increasingly common.

Recent studies furher highlight the rapid evolution of change detection methods toward more generalized and scalable frameworks. For instance, transformer-based and hybrid deep learning architectures have demonstrated improved capability in capturing long-range spatial-temporal dependencies and handling multi-sensor data integration, outperforming conventional CNN-based approaches in complex environments. These models enable more robust feature representation and improved generalization across regions and sensors, addressing key limitations of earlier methods.

In particular, recent works (Afaq and Koufi, 2026; Kaur and Afaq, 2024; Afaq and Manocha, 2022) demonstrate that integrating attention mechanisms, multi-scale feature extraction, and cross-modal fusion significantly enhances performance in time-series change detection tasks. These advancements indicate a clear shift from task-specific models toward more flexible and transferable architectures suitable for large-scale climate monitoring applications. This synthesis in Table 3 highlights that there is no universally optimal method for climate-relevant detection. Instead, each category offers a distinct trade-off between data requirements, achievable accuracy, transparency, and scalability. The framework presented here provides a structured basis for interpreting results across the diverse climate application domains reviewed in the following sections, and for selecting methods that are aligned with specific sensors, regions, and operational objectives. The application of these methods across specific climate domains and their performance in real-world scenarios are discussed in Section 4.

**Table 3:** Comparative synthesis of automated analysis techniques for climate change detection.

Method category/subtype	Typical data	Main climate applications	Typical metrics reported	Strengths	Limitations
Classical index differencing	Optical (Landsat, Sentinel-2), MODIS	Deforestation, crop loss, surface water change	OA, change magnitude	Very simple, fast, intuitive; minimal training data	Sensitive to seasonality and atmosphere; limited to strong signals
PCA / CVA-based methods	Multi-band optical, SAR stacks	General land cover and vegetation change	OA, Kappa, change magnitude	Multi-band, data-driven, unsupervised	Limited interpretability; noise sensitivity
Post-classification comparison	Any classified imagery	LULC transitions, deforestation, urbanization	OA, Kappa, transition matrices	Class-specific change; intuitive outputs	Error propagation; classification-dependent
Object-based approaches (OBIA/GEOBIA)	High-resolution optical, SAR	Urban growth, parcel-level LULC, coastal change	OA, F1, class accuracy	Uses shape and context; effective at high resolution	Segmentation sensitive; complex workflows
Supervised ML (RF, SVM, boosting)	Optical, SAR, DEM, indices	LULC mapping, deforestation, flood susceptibility	OA, F1, Kappa, AUC	Robust, interpretable, moderate data needs	Feature engineering required; limited temporal modeling
Unsupervised ML (K-means, GMM)	Optical/SAR feature stacks	Anomaly detection, pre-segmentation	Cluster purity, silhouette score	No labels required; exploratory analysis	Hard to map clusters to physical change
CNN-based classification	Optical/SAR image patches	LULC classes, burned areas, built-up detection	OA, F1, class accuracy	Learns spatial features automatically	Limited spatial context; boundary artifacts
CNN/U-Net segmentation	Optical, SAR, multi-sensor	Deforestation, flood mapping, snow/ice extent	OA, F1, IoU, Dice	Pixel-level maps; strong spatial accuracy	Label-hungry; computationally intensive
Siamese deep change detection	Bi-temporal image pairs	Binary and multi-class change detection	OA, F1, Kappa	Explicit change modeling; robust to illumination	Needs paired labels; sensitive to domain shift
Spatio-temporal DL (ConvLSTM, transformers)	Multi-year, multi-sensor time series	Gradual degradation, phenology, hazard evolution	F1, IoU, RMSE, trend error	Captures temporal dynamics; early detection	High data and compute demands; complex training

## 4 Climate applications of image processing

AI-based methods are now widely applied across multiple climate related domains, where they support systematic monitoring, attribution, and risk assessment using time-series remote sensing data. Different climate impact themes impose distinct requirements in terms of spatial resolution, revisit frequency, sensor modality, and algorithm design. This section organizes the literature by major climate application areas and summarizes how classical, machine learning, deep learning, and spatio-temporal methods are used in each domain, along with their typical strengths, limitations, and performance characteristics (Farooq and Manocha, 2024; Bai et al., 2023; Lu and Weng, 2020).

### 4.1 LULC and deforestation

LULC dynamics are among the most direct and visible manifestations of climate-relevant change, influencing carbon fluxes, surface albedo, evapotranspiration, and habitat continuity. Deforestation, agricultural expansion and intensification, afforestation and reforestation, urban growth, and land degradation all modify the exchange of energy, water, and greenhouse gases between the land surface and atmosphere, feeding back on local and regional climate. time-series image analysis using remote sensing has therefore become a core tool for quantifying the rate, spatial pattern, and drivers of LULC change in support of climate mitigation initiatives, adaptation planning, and ecosystem conservation (Mancino et al., 2025; McGregor et al., 2024; Delgado et al., 2025; Lahane et al., 2025).

LULC change detection approaches are effective in capturing large-scale transitions such as deforestation and urban expansion, particularly in regions with consistent temporal data availability. However, detecting subtle transitions such as forest degradation or mixed land-use systems remains challenging, especially in heterogeneous landscapes. At moderate spatial resolutions, for example with Landsat imagery, threshold-based index differencing and post-classification comparison can reliably detect large and abrupt transitions, such as clear-cutting or conversion from forest to cropland, especially when seasonal variability is controlled through compositing and phenology-aware date selection (Tesfaye et al., 2024; Huang et al., 2021; De and Barman, 2021; Shah et al., 2020).

More recent studies increasingly employ machine learning models such as Random Forests and gradient boosting trained on multi-sensor feature stacks. These feature sets often include spectral indices, textural descriptors, temporal statistics, and topographic variables, enabling improved discrimination between similar land-cover classes and more nuanced transitions, including forest degradation, shifting cultivation, and mixed agroforestry systems. Such models provide robust performance across heterogeneous landscapes and can produce variable-importance measures that support interpretability and driver analysis (Babitha et al., 2024; Şimşek, 2025; Gurjar et al., 2025).

For example, (Delgado et al. 2025) modeled land use and land cover changes in tropical coastal ecosystems using remote sensing and machine learning techniques, demonstrating the effectiveness of predictive modeling in capturing future land transition patterns. Their results highlight the potential of integrating temporal satellite data with data-driven models for long-term environmental monitoring.

Deep learning methods, particularly CNN-based segmentation networks and Siamese change-detection architectures, further improve boundary delineation and sensitivity to small or fragmented patches. They enable precise mapping of narrow deforestation fronts, complex landscape mosaics, and peri-urban growth patterns, especially when high-resolution optical or fused optical-SAR imagery is available (McGregor et al., 2024; Babitha et al., 2024). Spatio-temporal approaches based on image composites or index trajectories extend these capabilities by distinguishing permanent land-cover conversion from temporary disturbances, such as selective logging or seasonal agriculture, and by identifying breakpoints and trends in vegetation cover under combined land use and climate pressures (Miller et al., 2024; Huang et al., 2021).

Together, these techniques are enabling LULC and deforestation monitoring systems to evolve from static, infrequently updated maps to dynamic, routinely refreshed products that capture both the extent and temporal evolution of land-cover change. When combined with standardized accuracy metrics and uncertainty estimates, such products support climate reporting, mitigation program verification, scenario analysis, and evidence-based policy design.

While deep learning methods provide higher spatial accuracy and improved detection of fragmented and small-scale changes, their performance is highly dependent on the availability of large, well-annotated datasets. In contrast, classical and machine learning approaches remain more robust in data-scarce environments but struggle with subtle or heterogeneous transitions. This highlights a trade-off between accuracy and scalability, particularly in regions with limited ground truth data.

### 4.2 Urbanization and LST/urban heat

Urbanization is a key driver of climate-relevant change, reshaping land cover, surface energy balance, and local hydrometeorological regimes, with LST and urban heat islands emerging as critical indicators of impact. As natural and agricultural surfaces are replaced by impervious materials such as asphalt, concrete, and rooftops, changes in albedo, thermal inertia, and evapotranspiration lead to systematically higher surface and near-surface air temperatures in cities compared to surrounding rural areas, exacerbating heat stress, energy demand, and public health risks. Remote sensing-based image processing provides a consistent framework for mapping both the spatial footprint of urbanization and its thermal consequences, using multispectral optical data to delineate built-up areas and thermal infrared measurements to retrieve LST fields (Vahid and Aly, 2025; King et al., 2021; Xiong et al., 2021; Roy et al., 2020).

Classical approaches to urbanization and urban heat monitoring often rely on indices such as the Normalized Difference Built-up Index (NDBI), bare-soil indices, and vegetation indices, combined with thresholding or simple classifiers to separate built-up from non-built-up surfaces. LST is typically retrieved from thermal bands using radiative transfer corrections and surface emissivity estimates. These methods can reveal long-term urban expansion and associated warming patterns, but they may struggle with complex peri-urban mosaics and strong intra-urban heterogeneity (King et al., 2021; Xiong et al., 2021).

For example, (King et al. 2021) analyzed urban heat island effects using multi-temporal Landsat imagery and image differencing techniques, revealing significant temperature increases associated with urban expansion. This study illustrates how thermal remote sensing can be used to link land cover change with climate-induced thermal impacts.

Machine learning and deep learning approaches improve both urban land-cover discrimination and the interpretation of thermal patterns by integrating spectral, textural, and contextual features. For example, Random Forest and gradient boosting classifiers can map detailed urban structure classes, while CNN-based segmentation networks distinguish between dense built-up zones, low-rise residential areas, industrial surfaces, and urban vegetation, each with distinct thermal signatures. Joint analysis of LULC and LST time series enables separation of temperature changes related to land conversion from those driven by broader climate trends, supports quantification of urban heat island evolution across seasons and years, and allows evaluation of adaptation measures such as urban greening and reflective roofing. As a result, integrated urbanization LST products increasingly serve as input to urban climate models, heat-risk assessments, and climate-sensitive land-use planning.

Although deep learning and multi-feature machine learning approaches improve urban classification and thermal pattern analysis, distinguishing climate-driven temperature changes from land-use-induced effects remains challenging. This limitation complicates attribution of urban heat island dynamics solely to climate change, indicating the need for integrated modeling frameworks combining remote sensing with climatic variables.

### 4.3 Hydrology: lakes, rivers, floods, droughts

Hydrological systems are highly sensitive to climate variability and change, with impacts expressed through altered precipitation patterns, snow melt timing, evapotranspiration, and human water use. These drivers translate into changes in lake and reservoir levels, river discharge, wetland extent, flood frequency and severity, and intensity of drought. Earth observation data provides a unique synoptic and repeatable view to monitor such processes in river basins and regions by mapping the extent of surface water, inundation patterns, and moisture-related indicators over time. Optical multispectral data, SAR imagery, and in some cases thermal and altimetric measurements are combined through learning-based models to characterize both spatial extent and temporal dynamics of water systems (Yang et al., 2024; Khalil, 2025; Bhatt et al., 2022; Fang et al., 2021).

For lakes and reservoirs, classical image processing methods often apply water indices such as NDWI or Modified NDWI, or threshold SAR backscatter, to delineate open water, followed by time-series analysis to quantify area change linked to climate forcing and water management (Khalil, 2025; Fang et al., 2021). Rivers and wetlands are mapped using similar indices, frequently augmented with object-based segmentation or SAR-based classification to better resolve narrow channels and vegetated floodplains, particularly in persistently cloudy regions.

Flood mapping has become one of the most mature applications of image-based hydrological monitoring. Pre-, during and post-event optical and SAR scenes are processed using thresholding, index-based rules, supervised machine learning, or deep learning segmentation networks to produce high-resolution inundation masks. These products support emergency response, damage assessment, and calibration of hydraulic models (Yang et al., 2024; Bhatt et al., 2022). For drought assessment, indices that combine vegetation condition and LST, along with anomaly-based time-series indicators, are integrated with machine learning or temporal models to detect moisture stress, soil dryness, and agricultural impacts (Deo et al., 2020; Fang et al., 2021).

For example, (Bhatt et al. 2022) applied SAR-based thresholding techniques for flood inundation mapping, achieving high accuracy even under cloud-covered conditions. Their work demonstrates the importance of radar data in improving reliability of flood detection in operational scenarios.

Machine learning and deep learning are increasingly underpinning these hydrological applications. Random Forest and gradient boosting models trained on spectral, textural, and terrain features can robustly classify water presence, flood probability, and drought severity across heterogeneous landscapes. CNN and U-Net-type networks refine flood and permanent water masks, capturing complex shorelines and fragmented water bodies, while SAR-driven deep models enable reliable mapping under cloud cover and at night. Spatio-temporal models, including ConvLSTM and temporal CNNs, further extend the capability by learning typical seasonal water dynamics and identifying anomalies associated with extreme floods or prolonged droughts (Yang et al., 2024; Bhatt et al., 2022; Deo et al., 2020). Together, these image-driven hydrological products provide frequent, spatially explicit indicators of water availability, hazard exposure, and the hydrological signatures of climate change.

Despite high accuracy in flood and water mapping using SAR and deep learning models, performance often declines in complex terrains and mixed land-water interfaces. Additionally, drought detection remains indirect and dependent on proxy indicators such as vegetation indices, limiting the precision of climate impact assessment in hydrological systems.

### 4.4 Coasts and mangroves, shoreline and erosion

Coastal zones lie at the interface between land and ocean and are among the regions most exposed to climate-driven hazards, including sea-level rise, storm surges, and changing wave and sediment regimes. These pressures appear as shoreline retreat or advance, beach and dune reconfiguration, saltwater intrusion, and degradation or loss of protective ecosystems such as mangroves and salt marshes. Remote sensing-based image processing provides a synoptic and repeatable means to monitor such changes, supporting coastal risk assessment, shoreline management, and nature-based adaptation strategies. Medium- to high-resolution optical imagery is widely applied to delineate shorelines, intertidal zones, and vegetated coastal habitats (Vasquez et al., 2024; Tao et al., 2021; Wu et al., 2018), while SAR imagery contributes under cloudy conditions and for mapping inundation and structural change.

Classical shoreline monitoring approaches extract the land-water boundary from single scenes or multi-temporal composites using spectral thresholds, water indices, or edge-detection filters, and then quantify shoreline displacement by comparing derived lines across dates (Tao et al., 2021; Jensen, 2020; Wu et al., 2018). For mangroves and other coastal vegetation, spectral indices combined with supervised classification or object-based analysis are commonly used to distinguish mangroves from other land-cover classes and to map gains, losses, and degradation. For example, (Vasquez et al. 2024) reviewed mangrove cover change detection using Landsat imagery, highlighting the effectiveness of long-term satellite data in tracking coastal ecosystem degradation and recovery.

**Figure 6:**
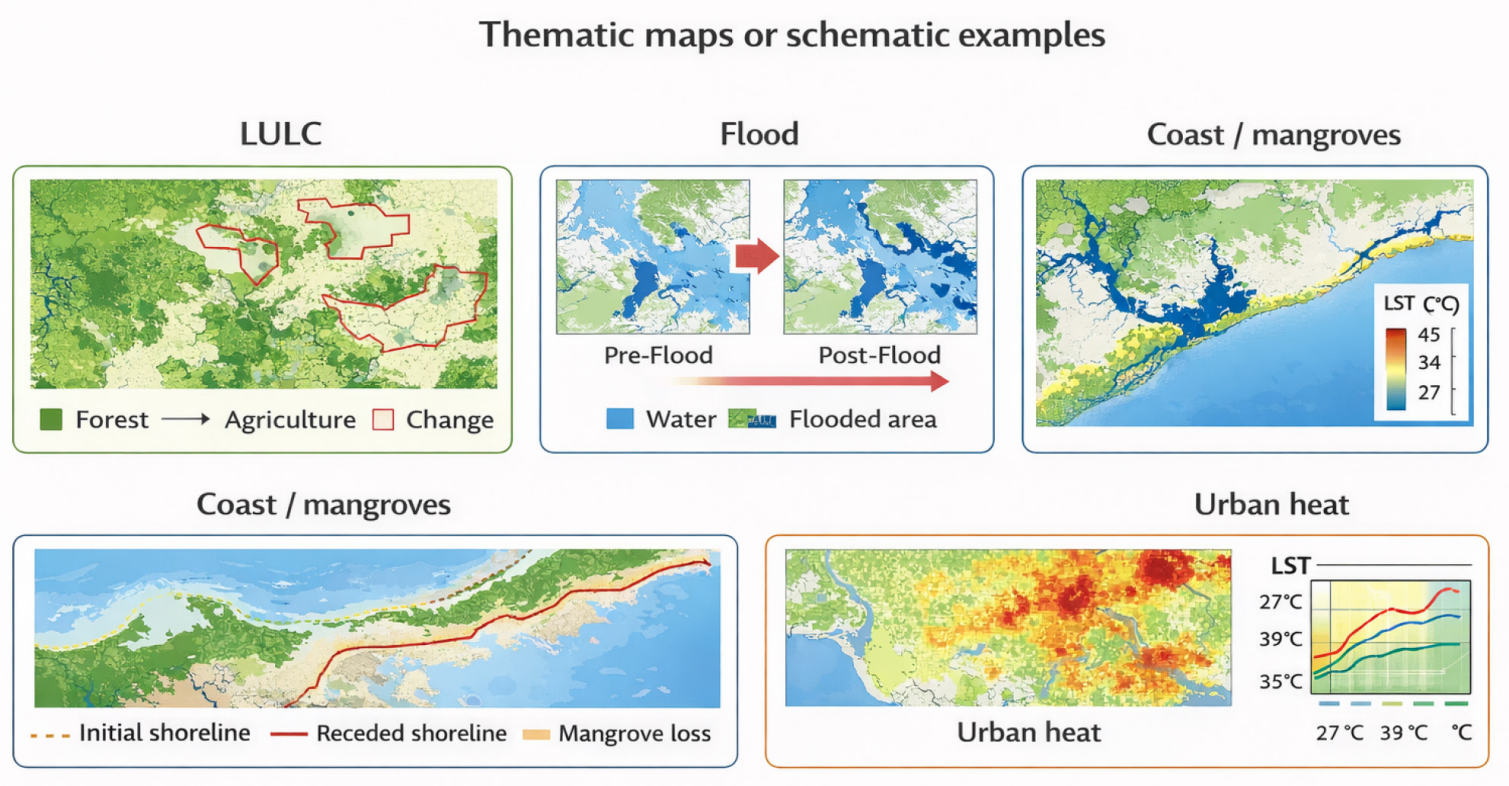
Thematic examples of climate change detection products from remote sensing, including LULC, floods, coastal and mangrove change, shoreline erosion, and urban heat (LST).

Machine learning models such as Random Forest classifiers improve class discrimination in complex coastal mosaics where water, wet soil, vegetation, and built-up areas coexist at fine spatial scales. Deep learning segmentation networks further enhance delineation accuracy for narrow shorelines, tidal creeks, and fragmented mangrove patches by jointly exploiting texture and spatial context. Time-series analyses based on dense satellite archives reveal both long-term erosional or accretional trends and short-term responses to extreme events, such as cyclone landfalls, followed by recovery trajectories. When combined with elevation data, wave climate indicators, and socio-economic layers, image-derived shoreline and mangrove change products support identification of coastal vulnerability hotspots and guide interventions including restoration, buffer-zone design, and engineered defenses (Tao et al., 2021; Wu et al., 2018). Representative thematic outputs and schematic examples across major climate application domains are shown in Figure 6.

While advanced segmentation models improve shoreline and mangrove delineation, their effectiveness is influenced by tidal variability, seasonal changes, and data availability. This introduces uncertainty in long-term change estimation, highlighting the need for consistent temporal normalization and multi-sensor integration.

### 4.5 Cryosphere: glaciers, snow, ice sheets

The cryosphere—comprising glaciers, seasonal snow cover, permafrost, river and lake ice, and the large ice sheets of Greenland and Antarctica–is one of the most climate-sensitive components of the Earth system. Its evolution serves both as a key indicator and a driver of climate change. Glacier retreat, thinning ice shelves, shrinking snow seasons, and altered melt dynamics have major implications for sea-level rise, regional water resources, and climate feedback processes. Multi-sensor data, combined with learning-based models, is indispensable for monitoring cryospheric change across vast and often inaccessible regions, providing consistent measurements of glacier extent, snow cover, melt onset, and surface conditions at spatial and temporal scales unattainable through ground observations alone (Shekhar et al., 2022; Chen and Zhang, 2014).

Optical multispectral imagery has long been used to map glacier outlines and seasonal snow cover using spectral indices and band ratios that exploit the strong reflectance contrast between snow or ice and surrounding terrain. Threshold-based and supervised classifications produce time series of glacier and snow extent that support estimation of retreat rates, snowline elevation shifts, and changes in accumulation and ablation zones. For example, (Shekhar et al. 2022) utilized Landsat time-series data and object-based change detection to monitor glacier retreat in the Himalayas, demonstrating the importance of temporal analysis in capturing long-term cryospheric dynamics. SAR data play a complementary role by enabling observation under cloud cover and polar night, and by supporting interferometric and feature-tracking methods to estimate glacier velocity and ice dynamics.

Thermal observations contribute to detecting melt onset and surface melt ponds, particularly on ice shelves and sea ice. Machine learning approaches improve class separation in challenging cases such as debris-covered glaciers and mixed snow-rock pixels, while deep learning segmentation networks increasingly automate glacier and snow mapping across large archives with improved robustness to shadows, illumination variability, and complex topography. Spatio-temporal models further extend these capabilities by integrating multi-year image sequences to separate persistent trends from interannual variability and to detect anomalies associated with extreme melt seasons or rapid dynamic change. Image-derived cryospheric products generated through these methods underpin assessments of glacier mass loss, contributions to sea-level rise, seasonal water supply from snowmelt, and the evolving stability of ice sheets under continued warming.

Although deep learning enhances detection of glacier and snow dynamics, challenges remain in accurately mapping debris-covered glaciers and separating seasonal variability from long-term climate trends. This limits the reliability of automated approaches in complex cryospheric environments.

### 4.6 Ecosystems and biodiversity monitoring

Ecosystems and biodiversity are being reshaped by interacting pressures from climate change, land use, pollution, and invasive species, resulting in shifts in habitat extent and configuration, species distributions, phenology, and ecosystem functioning. Remote sensing-based image processing enables systematic observation of these changes across large and often remote regions, providing spatially explicit indicators that support conservation planning, ecosystem-based adaptation, and assessments of nature's contributions to people. Optical multispectral imagery is widely used to map and monitor habitats such as forests, grasslands, wetlands, coral reefs in shallow waters, and tundra, while SAR and LiDAR contribute information on vegetation structure, biomass, and inundation dynamics. Hyperspectral imagery further supports fine discrimination of vegetation types and functional traits (Gomez-Chova et al., 2020; Chen and Zhang, 2014).

Classical approaches typically rely on land-cover classification and vegetation indices to track changes in habitat extent and condition, including forest loss and regrowth, wetland degradation, and desertification. Machine learning classifiers and deep learning segmentation models are increasingly applied to multi-sensor datasets to delineate habitat types with higher thematic accuracy and to detect more subtle degradation signals, such as canopy thinning, encroachment, and fragmentation (Gomez-Chova et al., 2020), that do not appear as complete cover conversion.

Time-series analyses of vegetation indices and phenological metrics reveal climate-driven shifts in growing season timing, productivity, and drought response, which serve as proxies for ecosystem stress and resilience. More advanced frameworks integrate image-derived habitat and condition maps with species occurrence records and ecological models to infer biodiversity patterns, landscape connectivity, and potential range shifts under future climate scenarios. Although satellite imagery does not directly measure species richness, image processing and AI substantially enhance monitoring of the environmental context that underpins biodiversity, enabling earlier detection of ecosystem change hotspots and evaluation of conservation and restoration outcomes at climate spatial scales.

For example, (Tesfaye et al. 2024) applied machine learning models within Google Earth Engine to analyze land use changes, providing insights into landscape dynamics and their ecological implications.

While remote sensing provides valuable proxies for ecosystem monitoring, it does not directly capture species-level biodiversity, leading to indirect and sometimes incomplete assessments. This gap highlights the need for integration with ecological and field-based data for more comprehensive analysis.

### 4.7 Natural hazards and extremes: wildfire, storms, multi-hazard mapping

Natural hazards and climate extremes, including wildfires, severe storms, landslides, heatwaves, and compound events, are projected to intensify or shift under climate change, increasing risks to populations, infrastructure, and ecosystems. Image processing and AI applied to Earth observation data play a central role in detecting, mapping, and in some cases forecasting these hazards, as well as in constructing multi-hazard assessments that account for spatial overlap and cascading effects (VanExel et al., 2025; Yang et al., 2024; Bhatt et al., 2022).

For wildfires, optical and thermal sensors are used to detect active fire fronts and to map burned area and burn severity, while SAR contributes to assessing structural change and pre- and post-fire moisture conditions. Classical approaches based on thermal anomaly thresholds and spectral indices, such as the normalized burn ratio, are increasingly complemented by machine learning and deep learning segmentation models that better handle smoke, cloud contamination, and heterogeneous backgrounds. Time-series methods further support separation of fire-induced change from longer-term vegetation dynamics (VanExel et al., 2025; Wu et al., 2021).

Storm-related hazards, including tropical cyclones and severe convective systems, are monitored using combinations of cloud, precipitation, and wind products, together with SAR and optical imagery for mapping inundation, wind damage, and coastal impacts. Flood extent mapping using SAR and multispectral imagery, often with AI-based classifiers, has become a standard component of rapid impact assessment following extreme rainfall and storm events (Bhatt et al., 2022). Landslides and mass movements are detected through pre- and post-event image comparison using change detection algorithms, object-based approaches, and increasingly CNN or U-Net models trained on landslide inventories. For example, (Yang et al. 2024) applied machine learning techniques to assess flood risks using satellite imagery, demonstrating the effectiveness of AI-based approaches in climate risk modeling and disaster management.

Multi-hazard and susceptibility mapping frameworks integrate outputs from hazard-specific detection models with terrain, land cover, and climate predictors. Machine learning and ensemble approaches are frequently used to estimate the joint likelihood of multiple hazards affecting the same area and to identify compound-risk hotspots, such as wildfire-flood or storm-landslide sequences. By delivering timely, spatially detailed information on hazard occurrence, evolution, and interaction, image-based hazard and multi-hazard products derived from advanced image processing and AI support early warning, emergency response, risk modeling, and climate-resilient planning (Farooq and Manocha, 2024; Vahid and Aly, 2025).

Although AI-based methods significantly improve hazard detection accuracy and speed, their reliability in real-time applications is constrained by data latency, model generalization issues, and sensitivity to environmental noise. This poses challenges for operational early warning systems.

## 5 Evaluation, performance metrics, and reliability

Reliable evaluation is essential for comparing image processing and AI methods for climate change detection and for determining whether models are suitable for scientific and operational use. Because tasks range from binary change detection and multi-class land-cover mapping to probabilistic risk estimation and continuous-variable prediction, multiple complementary metrics are required to capture performance from different perspectives (Chughtai et al., 2021; Foody, 2002). This section summarizes commonly used evaluation metrics, discusses how they are applied across method families, and highlights key reliability and benchmarking challenges.

### 5.1 Common classification and detection metrics

Evaluation of image processing and AI methods for climate detection typically relies on a set of standard classification and regression metrics, each capturing different aspects of predictive performance. OA is the simplest measure, defined as the proportion of correctly classified pixels or objects over all samples. It provides a convenient global summary when class distributions are relatively balanced and the goal is broad map correctness (Chughtai et al., 2021). However, OA can be misleading in strongly imbalanced settings, such as change vs. no-change, flood vs. non-flood, or burned vs. unburned mapping, where a model that predicts mostly the majority class may achieve high OA while failing to detect critical minority classes.

To address this limitation, class-specific measures are widely reported. Producer's accuracy, equivalent to recall or sensitivity from the reference-data perspective, measures the fraction of reference samples of a class that are correctly detected, indicating how completely that class is captured. User's accuracy, equivalent to precision from the map perspective, measures the fraction of predicted samples of a class that are actually correct, indicating how reliable mapped areas of that class are. These metrics are particularly important in climate applications where omission and commission errors have different consequences across classes.

Derived from precision and recall, the F1-score, defined as their harmonic mean, is especially useful for binary and imbalanced problems such as change detection, water mapping, and burned-area extraction, because it penalizes models that perform well on only one of the two components (Chughtai et al., 2021). For segmentation and spatial change mapping tasks, IoU, also known as the Jaccard index, measures the ratio between the overlap of predicted and reference regions and their union. IoU, and its class-averaged form (mIoU), is stricter than F1 in penalizing boundary and extent mismatches and is widely used to evaluate pixel-wise change maps and hazard footprints (Shafique et al., 2022; Bai et al., 2023).

The Kappa coefficient adjusts observed agreement by the agreement expected by chance based on class frequencies and has historically been widely reported in land-cover and change-detection studies. However, its interpretive advantage over class-wise accuracies and F1 or IoU is debated, particularly under severe class imbalance. For probabilistic outputs and ranking-based models, the Area Under the Receiver Operating Characteristic Curve (AUC-ROC) summarizes the trade-off between true positive and false positive rates across thresholds and is useful when decision thresholds are variable or when comparing susceptibility and risk models independent of a fixed cutoff.

For regression-style outputs, such as predicted LST, soil moisture, biomass proxies, or water level estimates, continuous-error metrics are used. Root Mean Square Error (RMSE), Mean Absolute Error (MAE), and bias quantify the magnitude and direction of deviations between predictions and reference values. In practice, robust evaluation in climate applications combines multiple metrics: OA and Kappa for global overview, producer's and user's accuracies for class-level error structure, F1 and IoU for minority or critical classes, and AUC or regression error measures when models produce probabilities or continuous outputs. This multi-metric perspective is essential because missing relatively rare but high-impact changes can be more consequential than small errors in dominant background classes.

### 5.2 How different methods are evaluated

Different generations of methods have developed somewhat distinct evaluation practices, which complicates direct comparison across studies (Farooq and Manocha, 2024; Bai et al., 2023). Classical image processing approaches, including index differencing, PCA/CVA, and post-classification comparison, typically report OA and sometimes the Kappa coefficient (Hussain et al., 2013; Lu et al., 2004; Singh, 1989), derived from an error matrix based on sampled reference points or polygons (Foody, 2002). In many earlier land-cover and change-detection studies, evaluation is limited to OA, and occasionally producer's and user's accuracies per class, with limited attention to class imbalance or spatially explicit error structure.

Machine learning methods, such as Random Forests, SVMs, and boosting models, more frequently report class-wise metrics, including producer's and user's accuracies, precision, recall, and F1-scores. For susceptibility and probability mapping tasks, such as flood or landslide susceptibility, Area Under the ROC Curve (AUC) is also commonly provided, reflecting a stronger emphasis on performance for specific classes or risk levels rather than only global accuracy.

Deep learning studies, particularly those based on segmentation and change-detection networks, commonly adopt segmentation-oriented metrics such as IoU or mIoU and Dice/F1 scores, in addition to OA and sometimes Kappa. Many also report per-class F1 or IoU values for critical categories such as “change”, “water”, or “burned area”, which are often minority but high-impact classes in climate applications.

Across all three method families, significant inconsistencies hinder direct comparison. Threshold-based classical methods often select change thresholds heuristically or through scene-specific tuning (Bovolo and Bruzzone, 2007; Bruzzone and Prieto, 2002), with limited reporting of sensitivity to threshold choice. Machine learning and deep learning approaches may similarly rely on implicit or unreported probability cut-offs when converting soft outputs to hard labels. Class definitions also vary widely across studies: for example, “forest loss” may or may not include degradation or plantation conversion, and “urban” classes may aggregate or separate residential, industrial, and transport surfaces differently.

Evaluation domains differ substantially in spatial and temporal scope, ranging from small pilot sites to multi-regional studies. Some works rely on random hold-out samples within the same region, whereas others use more stringent spatial or temporal hold-outs. Reference data sources also vary, including manual interpretation, national maps, and global products, each with its own uncertainty characteristics (Foody, 2002). Consequently, two methods reporting similar metrics such as OA or F1-score may have been tested under very different conditions, making naive metric-based ranking unreliable. Across multiple studies, a clear performance trend can be observed across different methodological categories. Classical image processing approaches typically achieve overall accuracies in the range of 70–85%, particularly in scenarios involving high-contrast changes such as deforestation or water expansion. Machine learning methods, including Random Forests and Support Vector Machines, generally improve performance to approximately 80–90% by leveraging multi-dimensional feature spaces and handling non-linear relationships.

Deep learning approaches, especially CNN-based segmentation and Siamese architectures, frequently report higher accuracies in the range of 85–95%, along with improved F1-scores and IoU, particularly in complex and heterogeneous environments. Recent spatio-temporal and transformer-based models further enhance performance by capturing temporal dependencies, often demonstrating superior generalization across multi-sensor datasets. However, these gains are accompanied by increased computational requirements and dependence on large labeled datasets. These indicative performance ranges are summarized in Table 4, illustrating the trade-offs between accuracy, data requirements, and computational complexity across different method categories.

**Table 4:** Typical performance ranges of change detection methods.

Method Type	OA (%)	F1 / IoU	Notes
Classical methods	70–85	Low–Moderate	Sensitive to noise
Machine learning	80–90	Moderate	Feature-dependent
Deep learning	85–95	High	Data-intensive
Spatio-temporal DL	88–96	Very High	High compute cost

### 5.3 Challenges in comparing performance

These inconsistencies contribute to broader challenges that make cross-study performance comparison inherently difficult. Class imbalance is one of the most significant issues (Chughtai et al., 2021; Foody, 2002). In many climate tasks, such as change vs. no-change, flood vs. background, or burned vs. unburned mapping, the minority class occupies only a small fraction of pixels. Under these conditions, a method can achieve high OA and even moderate Kappa by favoring the majority class while performing poorly on the critical minority class. Studies vary in how they address imbalance, for example through balanced sampling or class weighting, and often report only aggregate metrics that obscure omission-commission trade-offs.

Domain shift is another major challenge. Models trained and evaluated in one region, season, or sensor configuration may not generalize to others because of differences in climate, land-use patterns, sensor characteristics, and preprocessing pipelines. Many reported evaluations rely on random splits within a single geographic area, which can substantially overestimate performance relative to tests on independent regions or time periods.

A further limitation is the lack of standardized, cross-domain benchmark datasets for climate detection (Shafique et al., 2022; Farooq and Manocha, 2024; Bai et al., 2023). Although benchmark datasets exist for selected tasks, such as building change detection or generic land-cover mapping, there is no universally accepted benchmark suite spanning multiple climate applications, sensors, and biomes that would enable rigorous side-by-side comparison of classical, machine learning, and deep learning methods under consistent conditions. Differences in preprocessing, label quality, spatial resolution, and annotation protocols can produce large metric differences unrelated to algorithmic quality.

Finally, overfitting to specific regions or time periods remains a persistent risk, particularly for complex deep learning models trained on geographically limited datasets. Without explicit spatial and temporal cross-validation, domain adaptation strategies, and testing on independent regions and years, reported metrics may reflect memorization of local patterns rather than true generalization ability (Zhu et al., 2017). This can lead to overly optimistic performance claims that do not hold under operational deployment in new environments.

### 5.4 Toward benchmarking and reliable deployment

A transition toward robust benchmarking and reliable deployment of image processing and AI methods for climate detection requires a more coordinated approach to datasets, metrics, and evaluation protocols. A central requirement is the development of shared benchmark datasets spanning multiple climate applications, including LULC change, deforestation, floods, coastal dynamics, cryospheric change, and urban heat, across multiple sensor modalities such as optical, SAR, and thermal imagery. These benchmarks should include carefully curated and openly available reference labels and be designed to reflect realistic challenges, including class imbalance, cloud contamination, mixed land-use patterns, and domain shift, rather than only idealized pilot sites. Training and test subsets should be spatially and temporally disjoint to support meaningful assessment of generalization performance.

Standardized evaluation protocols are equally important. These include agreed data splits or cross-validation strategies, a minimum required set of reported metrics, and transparent reporting practices. A core metric set should typically include OA, class-wise producer's and user's accuracies, F1-score and IoU for critical or minority classes, Kappa where appropriate, and regression error metrics for continuous targets. Protocols should also specify how probability thresholds are selected, how uncertainty is quantified, and how computational cost and model complexity are reported. Public, reproducible baseline implementations of representative classical, machine learning, and deep learning methods on benchmark datasets would provide stable reference points for objective comparison of new approaches (Zhu et al., 2017).

Reliable operational deployment further requires practices that bridge the gap between research prototypes and production systems. Models should be tested under varying preprocessing pipelines and sensor combinations to assess robustness, and evaluated on out-of-distribution regions and time periods to quantify sensitivity to domain shift. Uncertainty estimates and explainability tools should be incorporated so that end users can understand confidence levels and likely failure modes. Open-source code, documented workflows, and, where licensing permits, pretrained models support independent verification, regional adaptation, and long-term maintainability by agencies and practitioners.

Finally, integration of well-benchmarked methods into scalable processing environments, such as cloud-based geospatial platforms, enables continuous re-evaluation as new data arrive and environmental conditions evolve. Ongoing monitoring of model performance and periodic revalidation against updated reference data help ensure that climate related image analysis systems remain reliable components of climate monitoring, risk assessment, and decision-support infrastructures (Pickens et al., 2025).

## 6 Challenges to adoption and practical deployment

Despite rapid methodological progress, significant barriers remain to the widespread adoption and operational deployment of image processing and AI methods for climate detection. These challenges span data and annotation availability, model generalization and transferability, computational and operational constraints, interpretability and trust, and integration with climate and decision-support systems. Addressing these issues is essential for translating research advances into reliable, scalable climate monitoring services (Farooq and Manocha, 2024; Bai et al., 2023).

### 6.1 Data and annotation limitations

High-quality data and annotations are foundational for training and evaluating image processing and AI models, yet they remain one of the primary bottlenecks for climate deployment (Shafique et al., 2022; Bai et al., 2023; Zhu et al., 2017). Generating reliable ground truth for land-cover change, floods, shoreline shifts, glacier retreat, or ecosystem degradation typically requires expert interpretation of time-series imagery, field surveys, or integration of heterogeneous national and local datasets. These processes are time-consuming, expensive, and difficult to scale across regions and years.

In many parts of the world, particularly across the Global South, mountainous terrain, and politically unstable regions, systematic reference data are sparse or absent. Researchers are often forced to rely on *ad hoc* sampling, outdated maps, or crowd-sourced annotations, which may not meet the consistency and accuracy requirements of climate monitoring and reporting. Even where labels exist, they are frequently noisy or uncertain: manual delineations vary between interpreters, historical maps may be based on coarser imagery, and global products can exhibit systematic biases for certain land-cover classes or environmental conditions.

Climate change classes, such as recent deforestation, rare flood extents, new landslides, or emerging mangrove loss, are often highly imbalanced relative to stable background classes. This makes it difficult to assemble sufficiently large and representative positive samples for training modern deep learning models without extensive manual labeling effort. As a result, models may be biased toward majority classes and under perform on precisely the rare but high-impact events that matter most for climate applications (Shafique et al., 2022; Farooq and Manocha, 2024; Bai et al., 2023; Yu et al., 2024).

These data and annotation limitations constrain model performance and generalization and complicate fair comparison between methods, since different studies rely on labels of varying quality, resolution, and provenance. Addressing this bottleneck will require coordinated development of open, well-documented reference datasets, wider adoption of semi-supervised and weakly supervised learning strategies that can leverage partially labeled or noisy data, and improved labeling protocols and interactive tools that reduce both the cost and subjectivity of ground-truth generation.

### 6.2 Generalization and transferability

A central challenge for climate-oriented image analysis is ensuring that models trained in one context generalize reliably to others (Zhu et al., 2017; Yu et al., 2024). Remote sensing data exhibit strong domain shift across sensors (different spectral responses, resolutions, and noise characteristics), regions (biomes, land-use patterns, and topography), and seasons (phenology, illumination, and snow cover). As a result, models that perform well on a particular training area and time period often degrade when applied elsewhere. Classical methods partly mitigate this risk by relying on physically interpretable indices and thresholds, but even these typically require region-specific tuning. Machine learning and deep learning models, which can capture intricate local patterns, are often even more sensitive to distribution shifts.

For climate monitoring systems that must operate globally or across long time spans, limited transferability undermines trust and requires repeated retraining or recalibration whenever sensors change or applications expand to new regions. Addressing this issue involves domain adaptation and transfer learning strategies, careful construction of training datasets that span diverse environmental and sensor conditions (Yu et al., 2024), and evaluation protocols that explicitly test spatial and temporal out-of-distribution performance rather than relying only on random splits within a single area.

### 6.3 Computational and operational constraints

Even when algorithms are technically mature, computational and operational constraints can limit their adoption by agencies responsible for climate monitoring and risk assessment (Pickens et al., 2025; Vahid and Aly, 2025). High-resolution, multi-temporal imagery over large territories quickly reaches terabyte to petabyte scale, while many operational institutions lack access to large GPU clusters or elastic storage systems, especially in resource-constrained settings. Deep learning models in particular may require substantial computational resources for both training and inference, making frequent product updates or reprocessing of historical archives difficult.

Operational choices often involve trade-offs between cloud-based processing, which provides elastic resources but depends on connectivity, data transfer, and recurring service costs, and on-premise or edge deployment, which offers greater control but is constrained by local hardware and maintenance capacity. Operational workflows must also emphasize robustness and automation, including fallback strategies when input data are missing or models fail, as well as versioning and provenance tracking to support auditing and reproducibility. In practice, there is often a trade-off between methodological sophistication and operational feasibility, and slightly less complex models that are more stable and easier to maintain may be preferable for real-world climate services.

### 6.4 Interpretability and trust in climate context

Because climate-related decisions often carry high stakes for land-use policy, infrastructure planning, and disaster risk management, there is a strong need for interpretability and trust in image-based outputs (Gu and Zeng, 2024; Zhu et al., 2017). Many deep learning models operate as black boxes, making it difficult for scientists and policymakers to understand why a given area is flagged as deforestation, flood, or high risk, or how sensitive those predictions are to uncertainties in input data and preprocessing.

In climate applications, decision-makers require not only accurate maps but also the ability to relate detected patterns to physical processes, such as vegetation structure change, soil moisture variation, or snow melt dynamics, and to reconcile model outputs with domain knowledge and *in-situ* observations. Limited transparency can slow acceptance of AI-derived products, raise concerns about bias or systematic regional errors, and complicate regulatory or legal use.

Approaches to address this include model explanation techniques such as feature importance analysis, saliency and attribution maps, and concept-based explanations, as well as explicit uncertainty quantification and communication. Architectures that incorporate physical constraints or hybrid statistical-physical structure can further enhance interpretability. Trust building also depends on open validation, transparent documentation of limitations, and close involvement of domain experts in interpreting and refining climate indicators derived from imagery.

### 6.5 Integration with climate models and decision systems

Image-derived products deliver maximum value when they are effectively integrated into broader climate models, early warning systems, and decision-support frameworks. However, linking pixel-level change maps and indicators to these systems is non-trivial. Climate and impact models often operate at coarser spatial and temporal resolutions and use aggregated variables such as albedo, surface roughness, vegetation parameters, or water storage. Using remote sensing outputs consistently therefore requires careful spatial and temporal aggregation, scale harmonization, and uncertainty propagation.

Early warning systems for floods, droughts, heatwaves, and wildfires must combine image-based observations with forecasts, ground measurements, and socio-economic data streams in near real time, which demands interoperable data formats, standardized interfaces, and reliable update schedules. Decision-support tools for land-use planning, conservation, and climate adaptation require that image-derived outputs be translated into scenario-relevant metrics that are understandable to non-technical stakeholders.

Misalignment in spatial scale, time horizon, metadata standards, and institutional responsibilities can all hinder integration. Overcoming these barriers requires co-design between remote sensing, climate modeling, and decision-analysis communities, agreement on shared ontologies and metadata standards, and modular processing pipelines in which image analysis components connect to modeling and planning systems through well-defined interfaces and quality controls.

## 7 Future trends

The next generation of climate image processing and AI will be shaped by advances that aim to overcome current limitations in data scarcity, generalization, scale, and integration with broader climate services.

### 7.1 Foundation models and self-supervised learning for remote sensing

Large foundation models trained on extensive multi-sensor Earth observation archives using self-supervised objectives are expected to reduce dependence on densely labeled datasets and improve transferability across regions, sensors, and applications. By learning generic spatial-spectral-temporal representations of the Earth's surface, such models can be fine-tuned with relatively small task-specific datasets to perform deforestation detection, flood mapping, and urban change analysis in new geographic areas (Yu et al., 2024). This capability directly addresses domain-shift and label-scarcity challenges that currently limit operational climate applications.

Self-supervised and contrastive learning strategies that exploit temporal consistency, multi-view imagery, and cross-sensor co-registration are particularly well suited to climate use cases, where long time series and heterogeneous sensors are common but high-quality labels are limited. These approaches enable models to learn invariant and physically meaningful representations from large unlabeled archives before supervised adaptation to specific change-detection tasks (Yu et al., 2024; Zhu et al., 2017).

### 7.2 Multi-sensor and multi-resolution fusion at scale

Future climate monitoring systems will increasingly rely on systematic fusion of optical, SAR, thermal, hyperspectral, and ancillary geospatial datasets across a wide range of spatial resolutions, from meter-scale commercial imagery to kilometer-scale global products. Methods that can ingest asynchronous, partially missing, and differently resolved inputs and reconcile them into coherent indicators will provide more robust monitoring under cloud cover, polar darkness, and sensor outages.

Advances are expected both at the model level, through multi-branch and attention-based deep architectures, and at the data-engineering level, through standardized preprocessing and cross-sensor harmonization frameworks. Such fusion will be particularly important for coastal change under extreme events, cryospheric monitoring, and soil moisture or drought assessment, where no single sensor provides complete and reliable coverage under all conditions (Bovolo and Bruzzone, 2015; Ghamisi et al., 2017).

### 7.3 Near-real-time global monitoring

With expanding satellite constellations and cloud-based geospatial processing platforms, near-real-time global monitoring of key climate related changes is becoming increasingly feasible (Pickens et al., 2025). Emerging systems are expected to deliver frequently updated global products for land-cover and land-use change, deforestation fronts, surface water dynamics, flood and wildfire extent, snow and ice cover, and urban expansion.

Realizing this vision requires robust, fault-tolerant pipelines capable of continuous data ingestion, automated preprocessing, and scalable inference. It also requires mechanisms to monitor model performance over time and to update models as environmental conditions and sensor configurations evolve. Low-latency products will support early warning systems, humanitarian response, and rapid climate-risk screening, while consistent near-real-time archives will support long-term trend and attribution studies.

### 7.4 Coupling with climate and impact models

Future developments point toward tighter coupling between image-derived products and climate, hydrological, ecological, and socio-economic impact models. Remote sensing-based estimates of land-cover change, albedo, vegetation parameters, surface water extent, and snow and ice dynamics can be assimilated into Earth system and regional climate models to constrain simulations and improve predictive skill (Bône et al., 2023).

Conversely, outputs from climate and impact models can guide image-based monitoring by identifying projected hotspots of change or elevated risk where higher-resolution or higher-frequency analysis is most valuable. Achieving effective two-way coupling will require scalable data assimilation methods, consistent variable definitions across communities, and workflows that propagate uncertainty between observational and modeling components.

### 7.5 Open benchmarks, shared models, and operational services

The field is moving toward a more open and service-oriented ecosystem built around shared benchmarks, open-source implementations, and operational delivery platforms. Community-curated benchmark datasets covering multiple climate applications, together with shared baselines and public evaluation protocols, will support more transparent and comparable method development (Shafique et al., 2022; Farooq and Manocha, 2024; Bai et al., 2023; Zhu et al., 2017).

**Figure 7:**
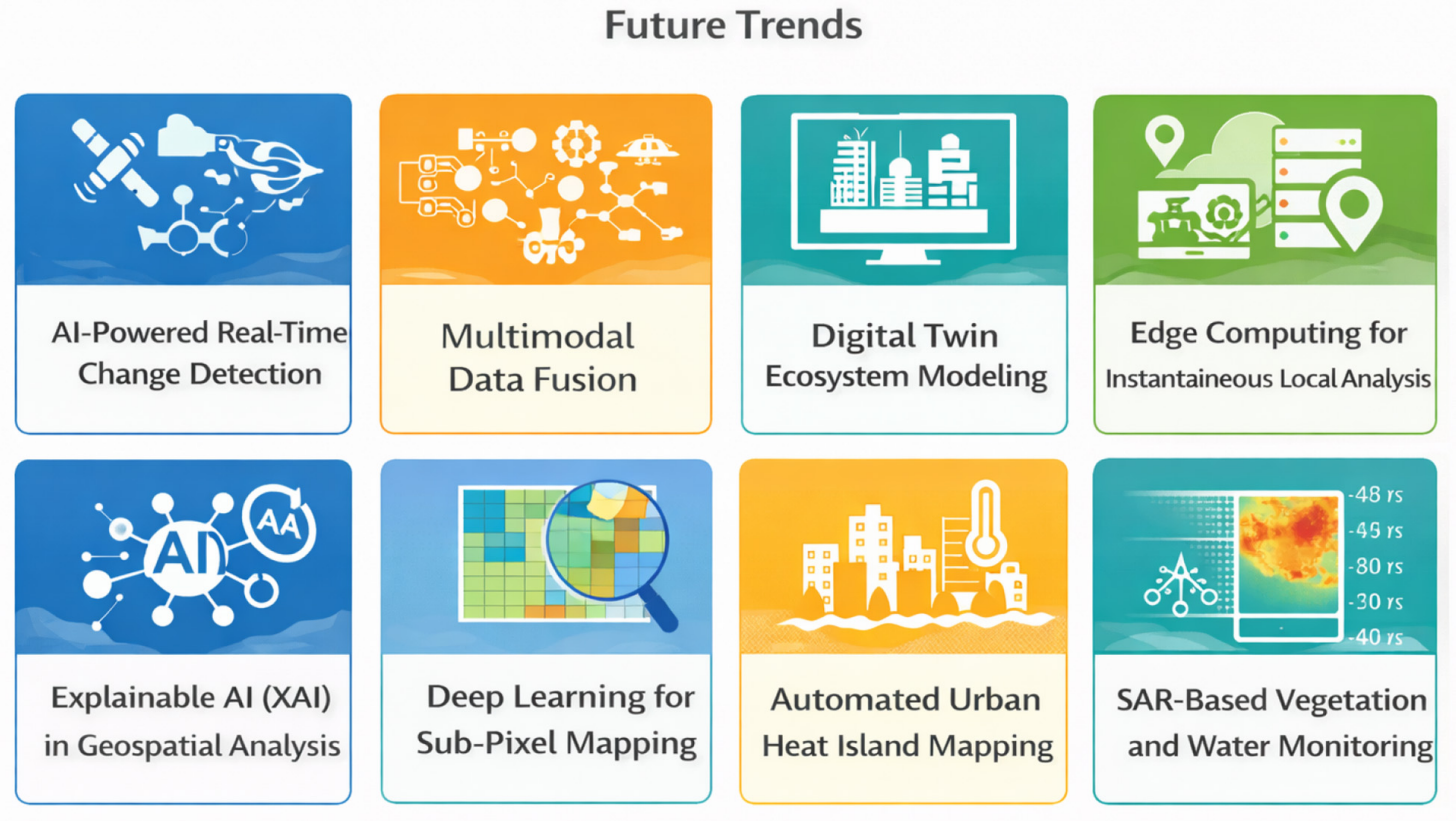
Emerging trends in image processing and AI for climate remote sensing, including foundation and explainable AI, multimodal fusion, digital twins, edge computing, and advanced change detection.

Open-source pipelines and pretrained models for classical, machine learning, and deep learning approaches will lower adoption barriers for agencies and practitioners, enabling adaptation to new regions and tasks. At the same time, operational web services and application programming interfaces delivering ready-to-use climate related layers, such as forest loss alerts, global surface water dynamics, and near-real-time flood or fire maps, will increasingly embed advanced AI-driven image analysis internally. Co-design with end users will be essential to ensure that such services are not only technically robust but also timely, interpretable, and aligned with climate adaptation, mitigation, and risk-management needs. Key emerging directions and technology trends shaping the next generation of climate image processing and AI systems are summarized in Figure 7.

## 8 Conclusion

Image processing and AI have transformed climate monitoring from a largely static and coarse view of the Earth into a dynamic, spatially detailed system capable of tracking land, water, coastal, cryospheric, and urban changes at regional to global scales. By combining multi-temporal satellite imagery with classical change detection, machine learning, and deep learning methods, current systems can map deforestation fronts, urban expansion, shifting surface water and floods, glacier retreat, snow cover variability, and emerging ecosystem degradation with far greater precision and temporal frequency than was previously possible (McGregor et al., 2024; Pickens et al., 2025; Babitha et al., 2024). These advances support not only improved description of past and ongoing changes but also near-real-time alerts and climate-risk assessments that inform adaptation and mitigation planning.

Among available techniques, classical image processing and index-based methods remain mature and widely used for long historical records and clear, high-contrast changes, particularly in land-cover and surface water mapping. Machine learning approaches such as Random Forests and gradient boosting are widely adopted for land-use and land-cover classification, hazard susceptibility mapping, and selected ecosystem and hydrological applications, offering a balance between accuracy, robustness, and interpretability. Deep learning methods, including CNN-based segmentation networks, Siamese change-detection architectures, and spatio-temporal models such as ConvLSTM and transformer-based approaches, now represent the state of the art for high-resolution change mapping, flood and wildfire delineation, shoreline and cryosphere segmentation, and multi-sensor fusion tasks (Shafique et al., 2022; Bai et al., 2023; Chen et al., 2020), especially where sufficient labeled data and computational resources are available. These approaches are most operationally mature in domains with large training datasets and strong demand, including deforestation monitoring, global land-cover mapping, flood and fire detection, and urban change analysis.

Despite substantial progress, important gaps remain in data availability, methodology, evaluation, and system integration (Farooq and Manocha, 2024; Zhu et al., 2017). High-quality and consistent annotations are still scarce for many regions and climate-sensitive processes, limiting model training and fair comparison, while domain shift across sensors, regions, and seasons continues to challenge generalization and reliable deployment. Evaluation practices remain heterogeneous, with varying metrics, thresholds, class definitions, and test protocols, making it difficult to benchmark methods or quantify trade-offs for rare but high-impact classes such as change, flood, or burned area. Future progress will depend on comprehensive open benchmark datasets and standardized evaluation protocols, wider adoption of foundation models and self-supervised learning to leverage unlabeled archives, more interpretable and uncertainty-aware model designs, and tighter coupling between image-derived products, climate and impact models, and decision-support systems. Addressing these needs will be essential for image processing and AI to deliver trustworthy, scalable, and operational climate intelligence for future adaptation and mitigation efforts.
